# Tuning the Polymorphism of the Anti-VEGF G-rich V7t1 Aptamer by Covalent Dimeric Constructs

**DOI:** 10.3390/ijms21061963

**Published:** 2020-03-13

**Authors:** Claudia Riccardi, Domenica Musumeci, Chiara Platella, Rosa Gaglione, Angela Arciello, Daniela Montesarchio

**Affiliations:** 1 Department of Chemical Sciences, University of Naples Federico II, Via Cintia 21, I-80126 Napoli, Italy; claudia.riccardi@unina.it (C.R.); domenica.musumeci@unina.it (D.M.); chiara.platella@unina.it (C.P.); rosa.gaglione@unina.it (R.G.); angela.arciello@unina.it (A.A.); 2 Institute of Biostructures and Bioimages (IBB), CNR, Via Mezzocannone 16, I-80134 Napoli, Italy; 3 National Institute of Biostructures and Biosystems (INBB), 00136 Rome, Italy

**Keywords:** aptamers, G-quadruplexes, V7t1, covalent dimers, biophysical characterization, VEGF_165_

## Abstract

In the optimization process of nucleic acid aptamers, increased affinity and/or activity are generally searched by exploring structural analogues of the lead compound. In many cases, promising results have been obtained by dimerization of the starting aptamer. Here we studied a focused set of covalent dimers of the G-quadruplex (G4) forming anti-Vascular Endothelial Growth Factor (VEGF) V7t1 aptamer with the aim of identifying derivatives with improved properties. In the design of these covalent dimers, connecting linkers of different chemical nature, maintaining the same polarity along the strand or inverting it, have been introduced. These dimeric aptamers have been investigated using several biophysical techniques to disclose the conformational behavior, molecularity and thermal stability of the structures formed in different buffers. This in-depth biophysical characterization revealed the formation of stable G4 structures, however in some cases accompanied by alternative tridimensional arrangements. When tested for their VEGF_165_ binding and antiproliferative activity in comparison with V7t1, these covalent dimers showed slightly lower binding ability to the target protein but similar if not slightly higher antiproliferative activity on human breast adenocarcinoma MCF-7 cells. These results provide useful information for the design of improved dimeric aptamers based on further optimization of the linker joining the two consecutive V7t1 sequences.

## 1. Introduction

The Vascular Endothelial Growth Factor (VEGF) family comprises different signaling cytokine proteins involved in both vasculogenesis and angiogenesis processes [1,2,3,4,5]. VEGF-A, or simply VEGF, is the founding member of this family and represents a potent growth cytokine, able to stimulate proliferation, migration and formation of endothelial cells [6]. Its most abundant known isoforms are VEGF_165_ and VEGF_121_ [7,8,9], which play prominent roles in pathological angiogenesis and vascularization of a large variety of solid tumors, and thus represent valuable biological targets for effective anticancer drugs [10]. The receptor binding domain is common to both VEGF_165_ and VEGF_121_, but only VEGF_165_ has also a heparin-binding domain, thus showing a more relevant biological activity than the other isoforms [7,8,9].

Using Systematic Evolution of Ligands by EXponential enrichment (SELEX) strategies, various nucleic acid-based aptamers [11] have been identified against this target, such as pegaptanib sodium, an RNA aptamer, component of Macugen, approved by the FDA for the treatment of age-related macular degeneration (AMD) pathology [12,13].

Aiming at the development of VEGF inhibitors as anticancer agents, Nonaka et al. [14] identified the DNA aptamer Vap7, which by subsequent optimization was truncated to evolve a 25-mer, named V7t1, with the sequence ^5′^TGT GGG GGT GGA CGG GCC GGG TAGA^3′^ [14]. This G-rich aptamer proved to bind with high affinity both VEGF isoforms, showing increased affinity for VEGF_165_ compared to Vap7 [14], and high in vitro antiproliferative activity on several cancer cell lines [15].

The intriguing biological properties showed by V7t1 have stimulated structural studies to better understand the effective conformations of this aptamer and thus design improved analogues. However, due to its marked polymorphism, no definitive structural data could be obtained on this VEGF-targeting aptamer, in any case investigated mainly in K^+^-rich solutions [16]. In this frame, to better understand the main features of the unmodified aptamer, and particularly its bioactive conformation, still largely unknown, our group investigated this aptamer in a Na^+^-rich medium (25 mM HEPES, 150 mM NaCl, pH = 7.4) [17], which better mimics the saline conditions of the extracellular environment in which VEGF targeting should occur.

These recent investigations provided new insights on this aptamer, which, in the tested Na^+^-containing buffer, showed a behavior dramatically influenced by the sample preparation procedure [17]. Indeed, V7t1 samples not subjected to annealing (i.e., directly dissolved in the selected buffer solution without any prior thermal treatment) formed in solution mainly dimeric parallel G4 structures, as evidenced by native polyacrylamide gel electrophoresis (PAGE), size exclusion chromatography (SE-HPLC), dynamic light scattering (DLS) and circular dichroism (CD) analyses. In contrast, when the V7t1 sample was subjected to annealing procedures, the aptamer significantly rearranged to give essentially monomeric G4 structures of mixed topologies [17]. Notably, electrophoretic mobility shift assay (EMSA) experiments unambiguously demonstrated that only the dimeric species formed in the not-annealed V7t1 samples were able to efficiently bind VEGF_165_, while the monomeric V7t1 species did not bind the protein under the same conditions [17].

Our results definitively proved that the protein showed a marked preference for dimeric G4 species also when the monomolecular species were concomitantly present in solution, reasonably due to multivalency effects, further favored by the fact that VEGF_165_ is a homodimeric protein [3,4].

The great affinity of VEGF_165_ for bivalent aptamers was indeed already demonstrated. In fact, Nonaka et al. also identified a mutant of V7t1, called 3R02 (sequence: ^5′^TGT GGG GGT GGA CTG GGT GGG TAC C^3′^), by three rounds of an in silico maturation [18]. Starting from this aptamer, they designed a bivalent derivative by connecting two 3R02 sequences through a 10-mer thymine linker which showed a ca. 10-fold higher affinity for the protein with respect to the monomeric 3R02 [18].

Furthermore, the same research group prepared a targeted construct for the heparin binding site of the homodimeric VEGF protein made of two identical anti-VEGF DNA-based aptamers (VEa5, with the sequence ^5′^ATA CCA GTC TAT TCA ATT GGG CCC GTC CGT ATG GTG GGT GTG CTG GCC AGA TAG TAT GTG CAA TCA^3′^). In this case the best results in terms of K_d_ values were obtained when no linker was introduced, while the presence of a linker containing from 10 to 20 T residues proved to be detrimental to the protein binding [19].

More recently, Manochehry and coworkers studied the affinity for VEGF_165_ of a series of dimeric aptamers based on the covalent connection of two heterodimeric or homodimeric DNA sequences using thymidine-based linkers of different length [20]. Among the dimeric derivatives of this series, the highest affinity was exhibited by the homodimer containing two consecutive sequences of the aptamer known as SL2-B (^5′^CAA TTG GGC CCG TCC GTA TGG TGG GT^3′^), able to recognize the heparin-binding domain [21] with *K*_d_ values in the nanomolar range [20]. The overall conclusion of this work was that the affinity of a dimeric aptamer for a homodimeric protein cannot be predicted, since not always dimerization produced higher affinity for the protein.

These are not the unique literature examples of aptamer optimization obtained by dimerization of known aptamer sequences. Indeed, in the last decade a number of works described improved aptamers realized by engineering bivalent or multivalent analogues of the selected oligonucleotide, not only for VEGF, but also for thrombin [19,22,23,24,25,26,27], mIgM (B-cell receptor) [28,29], and other biologically relevant targets [30,31,32], so that this strategy can be considered among those of choice to increase the overall efficacy of selected aptamers [33,34,35].

In addition to therapeutic approaches, an increasing interest has been also devoted to the use of dimeric aptamers in sensing applications. In thrombin targeting, the dimerization approach has been exploited also to achieve a sensitive and highly efficient acoustic-based protein detection, constructing bivalent aptamers by immobilization of a thrombin binding sequence on a gold surface. Then, a partially complementary aptamer sequence was hybridized with the immobilized one [36].

In this scenario, and particularly starting from our recent findings on the enhanced affinity of dimeric vs. monomeric V7t1 towards VEGF_165_, we here investigated a focused set of covalent V7t1 dimers in order to identify novel anti-VEGF G-quadruplex-based aptamers with improved performance. In the absence of high-resolution structural data on aptamer/VEGF_165_ complexes, no rational design can be performed to select optimal linkers connecting the two aptamer sequences, and previous literature examples show that essentially a trial-and-error procedure was followed thus far. Therefore, we designed new dimeric V7t1 constructs introducing either all-thymidine or oligoethylene glycol-based linkers, following typical strategies used in aptamer dimerization and/or oligonucleotide bioconjugation [19].

By using several biophysical techniques, i.e., electrophoretic, chromatographic as well as spectroscopic methods, the conformational behavior of these V7t1 dimers was studied in comparison with V7t1, particularly analyzing the influence of the different linkers on aptamer structuring. The ability of these V7t1 dimers to bind VEGF_165_ was also investigated by EMSA experiments in order to evaluate possible differences among the studied analogues in terms of affinity towards the protein. Finally, in preliminary in vitro investigations, their cytotoxic activity on human breast adenocarcinoma MCF-7 cells was evaluated.

## 2. Results and Discussion

### 2.1. Design and Preparation of the Covalent V7t1 Dimers

Based on our recent findings that V7t1, when not subjected to annealing, tends to form dimeric G-quadruplex structures having higher affinity for VEGF_165_ than its monomeric species, we here studied covalently linked V7t1 dimers. As main objectives, we aimed at possibly enhancing the V7t1 bioactive form and making the aptamer conformational behavior not strictly dependent on the specific pre-treatment used, e.g., the annealing procedure.

With this general purpose, three covalent V7t1 dimers, here indicated as **bisV7t1T7**, **bisV7t1HEG2** and **bisV7t1TEG2D**, were ad hoc designed introducing proper connecting linkers with similar overall lengths but different chemical features (Table 1). In detail, in **bisV7t1T7** two tandem V7t1 sequences were connected through a T_7_ linker providing a 57-mer. The selected T_7_ linker connects the 3′-end of one V7t1 sequence with the 5′-end of the other, thus maintaining the 5′-3′ polarity throughout the dimeric oligonucleotide (Figure S1, Scheme I). This derivative was selected as a reference compound essentially following the approach previously explored by Nonaka et al. in the construction of the bivalent 3R02 analogue [18,37].

In the case of **bisV7t1HEG2**, two tandem V7t1 sequences, both in the 5′→3′ direction (Figure S1, Scheme I), were connected by a flexible linker consisting of two units of hexaethylene glycol (HEG).

Then, in the last studied system, named **bisV7t1TEG2D**, the 3′ extremities of two V7t1 sequences were connected so to include a 3′-3′ inversion of polarity site (Table 1 and Figure S1, Scheme II). In this case, the linker contains two triethylene glycol (TEG) residues connected to a central symmetric doubler (Figure S2a).

All the selected V7t1 dimers were prepared by solid phase oligonucleotide synthesis using phosphoramidite chemistry. In particular, for **bisV7t1TEG2D**, a CPG-^3′^symmetric doubler 2DNA (Figure S2a) was exploited for the simultaneous elongation of the V7t1 sequences using standard 3′-phosphoramidites, in an approach in which the couplings necessary for the oligonucleotide synthesis are halved [38]. Conversely, the CPG-^3′^dA^5′DMT^ (Figure S2b) was used as starting support for the assembly of both **bisV7t1T7** and **bisV7t1HEG2**, not including inversion of polarity sites. Then, HEG- and TEG-based spacer-cyanoethyl (CE) phosphoramidites (Figure S2c,d) were exploited to link two V7t1 sequences in **bisV7t1HEG2** and **bisV7t1TEG2D**, respectively.

All the designed linkers spanned similar lengths, in the range of 40–42 bonds, considered suitable to keep the extremities of two V7t1 sequences at a proper distance to allow their optimal folding [17].

In order to evaluate the effects of different saline conditions on the structuring ability of these aptamers, all the biophysical investigations were carried out in two different buffer solutions, containing a high content of Na^+^ (25 mM HEPES, 150 mM NaCl, pH = 7.4, here indicated as HEPES/Na^+^) or K^+^ (10 mM Tris, 100 mM KCl, pH = 7.1, here indicated as TRIS/K^+^) ions, respectively mimicking the extra- and intracellular environment. It is indeed well known that buffer composition, and particularly ion nature and concentration, can produce dramatic changes in the aptamers secondary structure, finally affecting their stability and activity [39,40,41,42].

The selected Na^+^-rich buffer mimes the blood salt composition and is indeed more appropriate for studying the structure-activity relationships of these aptamers in the extracellular media, although the stability of the G4 module is generally decreased in these saline conditions. In addition, the VEGF_165_ protein was provided folded in this solution in which we had previously performed the key experiment demonstrating the preference of the protein for dimeric V7t1 [17].

Taking into account the different conformational behavior and VEGF binding properties of V7t1 if subjected to annealing or not, all the covalent V7t1 dimers were analyzed in both forms to verify in detail their behavior. Thus, the not-annealed samples (here abbreviated as N.A.) were obtained by simple dissolution of the required oligonucleotide amount in the selected buffer from a concentrated stock solution in water. The annealed samples (here indicated as A.) were in turn prepared by dissolving the oligonucleotides at the desired concentration in the selected saline solution, keeping them at 100 °C for 5 min and then leaving them to slowly cool at room temperature overnight.

### 2.2. Gel Electrophoresis Analysis

To further check the purity of the here studied V7t1 dimers, also assessed by HPLC and Matrix-Assisted Laser Desorption/Ionization (MALDI) data provided from the commercial suppliers, a 20% denaturing PAGE analysis was carried out, in comparison with the 25-mer V7t1 (Figure S3). In denaturing conditions, all the investigated oligonucleotides migrated as a single band on the gel, showing a retarded mobility for the covalent V7t1 dimers compared to V7t1, as expected for their higher molecular weights, respectively corresponding to a 57-mer for **bisV7t1T7** and to a 50-mer for **bisV7t1HEG2** and **bisV7t1TEG2D**.

**Figure 1:**
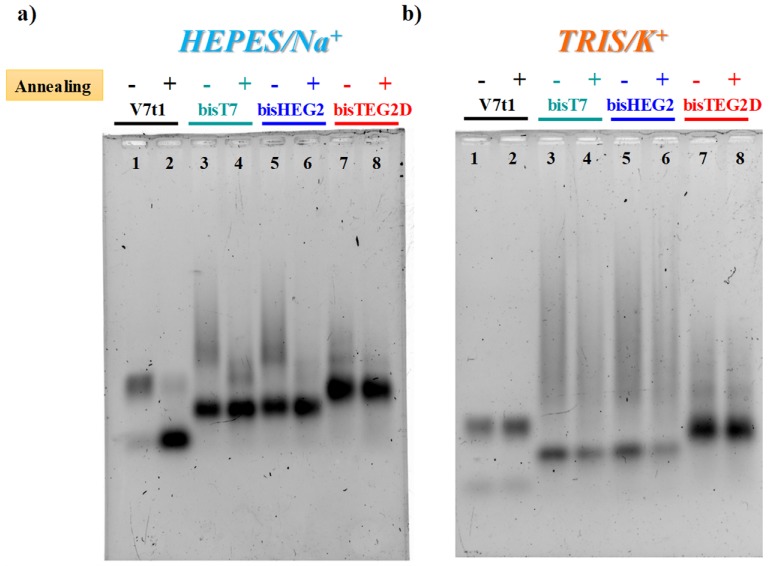
The 2% agarose gel electrophoresis under native conditions of V7t1 and its covalent dimeric analogues (here indicated as **bisT7**, **bisHEG2**, **bisTEG2D)** in both not-annealed (N.A.) (−) and annealed (A.) (+) form at 4 μM concentration in the selected HEPES/Na^+^ (**a**) and TRIS/K^+^ (**b**) buffer solutions. Gels were run at constant 60 V at r.t. for 2 h in Tris-Borate-EDTA (TBE) 1X as running buffer.

Then, agarose (Figure 1) and acrylamide (Figure S4) gel experiments in native conditions were also performed to characterize the selected V7t1 dimers in both N.A. and A. forms, as well as in different buffer solutions, in order to determine the number of species they form in solution.

In both gel assays, in the selected HEPES/Na^+^ buffer, all the investigated V7t1 dimers showed a detectable difference if in N.A. or A. forms, as in the case of V7t1 [17]. In particular, **bisV7t1T7** and **bisV7t1HEG2** in the N.A. samples migrated as two distinct bands with a marked difference in their electrophoretic mobility (Figure 1a and Figure S4a, lanes 3 and 5), indicative of very different structuring. Indeed, a band with the mobility expected for the dimeric aptamer was found, along with very large species (aggregates or higher order G4 structures) which moved as smeared bands in the agarose gel (Figure 1a, lanes 3 and 5) and hardly entered the polyacrylamide pockets (Figure S4a, lanes 3 and 5). For these samples, upon annealing in the Na^+^-rich medium, aggregate formation was sensibly reduced as clearly detectable in both gels (Figure 1a, lanes 4 and 6).

With respect to **bisV7t1T7** and **bisV7t1HEG2**, **bisV7t1TEG2D** behaved differently: its N.A. and A. samples migrated almost in the same manner, showing a predominant band with similar mobility as the other two covalent dimers, along with some less intense bands, better solved in the acrylamide-based gel, indicative of additional structures in solution, plausibly distinct conformations rather than large aggregates (Figure 1a and Figure S4a, lanes 7 and 8). Thus, in the Na^+^-containing saline conditions, the N.A. samples of **bisV7t1T7** and **bisV7t1HEG2** showed enhanced aggregation compared to **bisV7t1TEG2D**, while in their annealed counterparts aggregate formation was sensibly reduced.

Compared to the Na^+^-rich medium, completely different was the behavior observed in both gels for V7t1 in TRIS/K^+^ buffer (Figure 1b and Figure S4b), here analyzed for the first time. In addition, in these saline conditions, V7t1 showed the presence of two distinct bands corresponding to the monomeric and dimeric G4 structures found in HEPES/Na^+^ buffer [17], but without detectable differences between the N.A. and A. samples (Figure 1b and Figure S4b, lanes 1 and 2).

Accordingly, covalent V7t1 dimers migrated in an overall similar manner independently from the sample preparation procedure. Indeed, for all the covalent V7t1 dimers here investigated, two main bands were clearly distinguishable: one with higher electrophoretic mobility, corresponding to the expected covalent dimeric species, and a retarded one, reasonably associated to large aggregates or higher order G4 structures (Figure 1b and Figure S4b, lanes 3–8).

Notably, compared to **bisV7t1T7** and **bisV7t1HEG2**, **bisV7t1TEG2D** exhibited reduced aggregate formation with and without annealing, showing the expected covalent dimer as the main species (Figure 1b and Figure S4b, lanes 7 and 8). Overall, no sensible difference in terms of aggregate formation was found in the selected TRIS/K^+^ buffer between the N.A. and A. form of all the tested molecules, and for **bisV7t1TEG2D** analysed in the Na^+^-rich medium.

However, in both saline conditions, the bands corresponding to the covalent V7t1 dimers had a slightly increased mobility with respect to the dimer which V7t1 spontaneosly formed when dissolved in the selected buffer. Indeed, gel mobility of G4 structures is essentially affected by their conformation and compactness provided that their mass/charge ratio is quite similar. Since this is our case, PAGE results suggested a more compact structure for all the covalent V7t1 dimers, producing a faster migration on the gel [43,44].

Additionally, some differences (more marked in the acrylamide than in the agarose gel, Figure S4) could be detected among the studied analogues, suggesting that in both buffers the general trend of the V7t1 dimers mobility was: **bisV7t1HEG2** > **bisV7t1T7** > **bisV7t1TEG2D** > V7t1 dimer.

Taking into account that the used buffer solutions differ not only for the prevalent metal cations but also for the presence of different amines in solution, i.e., HEPES and TRIS, reported as possible DNA interactors [45], we further verified that the observed differences in the migration ability on the gels were effectively dependent on the metal cations composition. Thus, 2% agarose gels in native conditions were performed for V7t1 and its covalent dimers, in both N.A. and A. forms, in buffers solutions not containing the amines, i.e., 150 mM NaCl (pH = 7.4), as Na^+^-rich buffer and 100 mM KCl (pH = 7.3), as K^+^-rich buffer (Figure S5).

In both saline conditions, the migration ability of the oligonucleotides and the number of species they formed in solution was consistent with what previously observed in the selected HEPES/Na^+^ and TRIS/K^+^ solutions (Figure 1). These results—within the resolution limits of the technique—demonstrated that the aptamer structuring mainly depend on the prevalent ions in solution and on the sample preparation procedure (A. vs. N.A. form), in turn being negligible the contribution of the amines present in the used buffer solutions. This further corroborates the cognition that the nature and concentration of the metal cations in solution is the most crucial factor affecting the overall conformation of G-quadruplex structures [42].

On this basis, the amine-free Na^+^- and K^+^-rich buffers were not further investigated.

### 2.3. Size exclusion (SE) Chromatography Analysis

Size exclusion (SE)-HPLC recently emerged as an attractive and useful technique to study the different species formed by polymorphic G4s [46,47], often used in tandem with electrophoretic methods [17,48]. SE-HPLC analyses were here performed in order to further investigate the number and nature of the species formed by covalent V7t1 dimers.

In HEPES/Na^+^ buffer (Figure S6), as already observed in gel electrophoresis analysis and found for V7t1 [17], the behavior of covalent V7t1 dimers was dramatically influenced by the sample preparation procedure. Indeed, N.A. samples showed in all cases a predominant peak, at *t*_R_ ca. 9 min, corresponding to the expected value for a V7t1 dimeric form, accompanied by significant amounts of larger species with shorter retention times (*t*_R_ ca. 6 min). In addition, a very weak peak (*t*_R_ ca. 10 min) was present (Figure S6a). This retention time was quite different from that found in the same experimental conditions for monomeric V7t1 (*t*_R_ = 11.3 min) suggesting that it may be correlated to other V7t1 dimeric conformations. A slightly different behavior was found for **bisV7t1TEG2D** (Figure S6, red line); in this case, species with shorter retention times were not well separated from the predominant peak of the dimer, in line with PAGE results showing multiple retarded bands.

Conversely, all the A. samples eluted mainly as a single species with *t*_R_ of about 9 min. A very weak peak with longer elution times was also found for all the covalent V7t1 dimers, suggesting the presence in solution of small amounts of different conformations (Figure S6b).

When analyzed in the TRIS/K^+^ buffer solution (Figure S7), neither V7t1 nor its covalent dimers showed detectable differences on comparing their N.A. and A. samples. Indeed, V7t1 gave two well-separated peaks on the SEC column corresponding to dimeric and monomeric structures with the same overall abundance, with a marked predominance of the dimeric species (ca. 70%), no matter if the sample was subjected to annealing or not. In turn, the covalent V7t1 dimers showed multiple peaks with retention times in the 6–9 min range, confirming the presence of large aggregates, as well as of the main, expected species in different conformations (Figure S7).

The chromatographic data were well consistent with the electrophoretic results, confirming that in HEPES/Na^+^ buffer the species formed in solution by the here investigated covalent V7t1 dimers were sensibly influenced by the preparation method. On the contrary in the selected TRIS/K^+^ solution, the sample preparation procedure did not markedly affect the number and overall behavior of the species in solution. Notably, when the covalent V7t1 dimers were annealed in the Na^+^-rich medium, essentially one main species corresponding to the expected one was found, suggesting a net reduction of the polymorphism of these aptamers under these conditions, otherwise forming multiple species.

### 2.4. Spectroscopic Characterization and Singular Value Decomposition (SVD) Analyses of Covalent V7t1 Dimers Folding

#### 2.4.1. UV Analysis

The spectroscopic properties and the conformational behavior of the covalent V7t1 dimers were investigated by means of UV and CD spectroscopies in the selected buffer solutions.

In detail, for all the covalent V7t1 dimers here studied, UV thermal difference spectra (TDS) were obtained recording spectra at low and high temperatures (15 and 90 °C, respectively) for both N.A. and A. form at 2 μM concentration. Differential spectra—representing the spectral difference between the unfolded and the folded oligonucleotide—have peculiar patterns allowing identifying different nucleic acid secondary structures, and in particular G-quadruplexes [49,50,51].

In HEPES/Na^+^ buffer (Figure S8), almost all the analyzed compounds (both N.A. and A. samples) showed TDS profiles with maxima at ca. 257 and 270 nm, along with two minima at ca. 243 nm and 295 nm. The presence of a minimum at 295 nm associated with the maximum at 273 nm is diagnostic of a G4 structure [49]. However, this minimum was not very pronounced, as already observed for V7t1 analyzed under the same saline conditions [17], suggesting that probably not all the oligonucleotide is folded into a G4 and additional structures can be present in solution [49,50,51]. The minimum around 295 nm, in the case of A. **bisV7t1T7** and **bisV7t1HEG2**, was negligible, indicative of a low amount of G4 in these samples, perhaps due to a not complete unfolding/refolding of the structure at 90 °C. Slight differences between the covalent V7t1 dimers and, in the case of the same aptamer, between its N.A. and A. form, were found (Figure S8).

Conversely, in TRIS/K^+^ buffer solution no substantial difference was detected comparing each N.A. aptamer with its A. form: in all cases, the TDS profiles showed two maxima, at ca. 257 and 270 nm, along with a minimum at ca. 295 nm (Figure S9).

**Figure 2:**
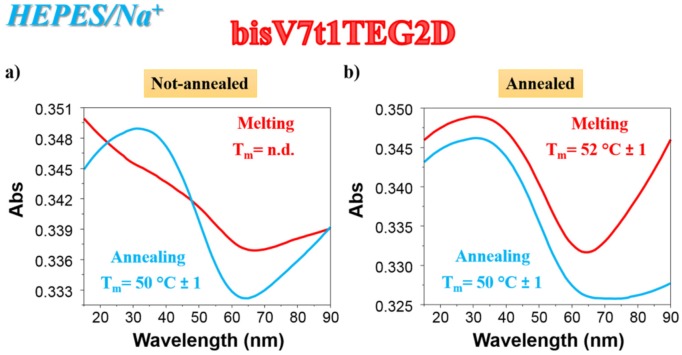
UV analysis on **bisV7t1TEG2D** at 2 µM concentration in the selected HEPES/Na^+^ buffer solution in both N.A. (**a**) and A. (**b**) form: overlapped UV-melting and UV-annealing profiles (red and light blue lines, respectively) recorded at 295 nm using a scan rate of 1 °C/min. n.d. = not determined.

As far as the UV thermal denaturation/renaturation experiments are concerned, no covalent V7t1 dimer showed clear sigmoidal profiles in the melting or in the annealing process monitored at 295 nm in both the selected saline conditions, but simply a progressive reduction of the absorbance on raising the temperature (data not shown). The only exception to this trend in the UV thermal profiles at 295 nm was **bisV7t1TEG2D** in HEPES/Na^+^ buffer solution: the N.A. sample showed a UV-melting curve with a detectable decrease of the absorbance upon increasing the temperature (Figure 2a, red line). Remarkably, the UV-annealing profile, recorded upon cooling down the samples from 90 to 15 °C, provided a sigmoidal curve with an apparent T_m_ value of ca. 50 °C, not superimposable to the corresponding melting curve (Figure 2a, light blue line).

Differently from the N.A. **bisV7t1TEG2D**, its A. sample showed sigmoidal denaturation pathways with almost superimposable melting/cooling profiles and only limited hysteresis, indicating that under the experimental conditions used (scan rate: 1 °C/min), the related processes were essentially reversible (Figure 2b). Monitoring the UV absorbance at 295 nm, apparent T_m_ values of 52 and 50 °C were respectively derived from the heating and cooling curves (Figure 2b, red and light blue lines, respectively), indicating the formation of quite stable G4 structures.

Aiming at verifying if some duplex structures were present in our samples, the UV thermal denaturation/renaturation experiments were registered also at 260 nm. In all cases (here reported for **bisV7t1T7** as a representative example, Figure S10) a progressive absorbance increase and decrease were respectively found during the heating and cooling experiments, but no net sigmoidal behavior was observed. Specifically, the melting curves provided only a general drift (with the sole exception of the A. bisV7t1T7), while the annealing profiles showed a clear plateau at high temperature and then a gradual reduction of the absorbance intensity (Figure S10), in all cases not providing evidence of the presence of significative duplex structures in the analyzed sample.

#### 2.4.2. CD Spectra and SVD Analysis

CD spectroscopy is a primary tool for the characterization of G4 structures/topologies [51], also particularly useful in the study of dimeric sequences [52]. Typical antiparallel G4 structures show positive Cotton effects with bands centered at ca. 290 and 240 nm, associated with a negative band at 265 nm. CD spectra of parallel G4 structures give positive and negative effects with bands centered at ca. 265 and 240 nm, respectively. In a typical hybrid G4 topology, a positive and a negative band at respectively 290 and 240 nm are observed [53,54,55,56,57,58,59,60]. Thus, in order to disclose the conformational properties and thermal stability of the G4 structures formed by the covalent V7t1 dimers, and also evaluate the effects of the linkers inserted between the two consecutive V7t1 sequences, CD spectra and CD thermal denaturation measurements were recorded.

**Figure 3:**
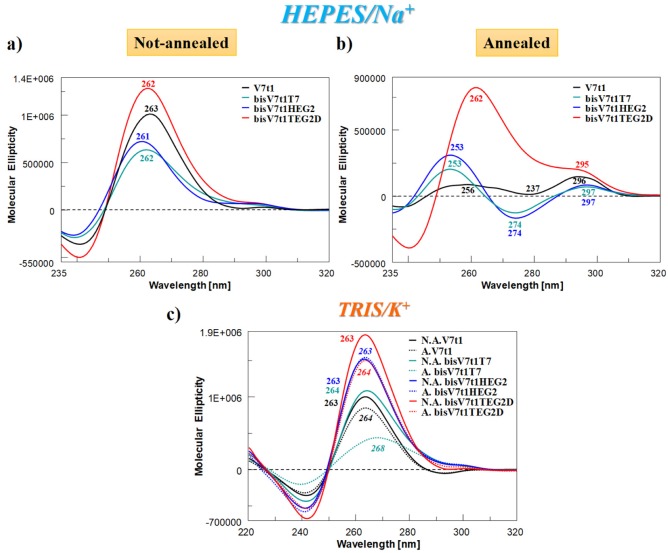
Overlapped circular dichroism (CD) spectra of V7t1 (black line) and covalent V7t1 dimers (green, blue and red lines, respectively for **bisV7t1T7**, **bisV7t1HEG2** and **bisV7t1TEG2D**), recorded at 15 °C and 2 µM concentration in the selected HEPES/Na^+^ (**a**, **b**) and TRIS/K^+^ (**c**) buffer solutions. All the investigated oligonucleotides were analyzed in both N.A. and A. form, reported respectively in panel a and b for HEPES/Na^+^ and as continuous and dashed lines in panel c for TRIS/K^+^ buffer solution.

The CD spectra at 15 °C of covalent V7t1 dimer—at 2 µM conc. in both N.A. and A. form and in both the selected solutions—are reported in Figure 3 in comparison with V7t1, analyzed under the same experimental conditions.

In HEPES/Na^+^ buffer, the spectra of the N.A. samples (Figure 3a) nicely showed that all the covalent dimers folded into G4 structures displaying a CD profile very similar to that of V7t1 (Figure 3a, black line). In detail, all the oligonucleotides showed a main positive band with a maximum centered at ca. 260 nm—consistent with a parallel G4 structure [56]—accompanied by a very weak band at ca. 295 nm, indicative of only very low amounts of other G4 conformations (Figure 3a).

Completely different was the behavior of the A. samples (Figure 3b). In the Na^+^-rich medium, both A. **bisV7t1T7** and **bisV7t1HEG2** displayed CD spectra [51,56,57] with two positive bands around 250 and 297 nm (with the latter one rather small with respect to the other) and one negative band centered at 274 nm (Figure 3b). These spectral features can be attributed to the coexistence in solution of multiple G4-conformations, as already observed for the annealed V7t1 sample [17]. Compared to the other A. samples, dramatically different was the CD spectrum of **bisV7t1TEG2D** (Figure 3b, red line), in which a prevalent CD signal centered at 260 nm was found, along with a detectable band centered at 295 nm. These spectral features revealed for **bisV7t1TEG2D** a main parallel G4 folding also after annealing, with only minor amounts of coexisting antiparallel or hybrid G4 topologies, however increased compared to the same N.A. sample.

As far as the samples in the TRIS/K^+^ buffer solution were concerned, in both N.A. and A. form (Figure 3c, continuous and dashed lines, respectively), the CD spectra were in all cases indicative of parallel G4 structures with a main band centered at ca. 260 nm. Differences were observed only in the intensity of the bands, except for A. **bisV7t1T7**, showing a positive band with a maximum at 268 nm (Figure 3c, dashed green line).

In order to verify the coexistence of multiple G4 topologies and their relative amount in the analyzed samples, the recorded CD spectra were also processed by singular value decomposition (SVD) analysis by exploiting the software recently developed by del Villar-Guerra et al. [60]. By using a constrained nonlinear least-squares fitting, they proposed a rapid and powerful tool to obtain quantitative information about the secondary and tertiary structure parameters from the CD spectra of a selected G-rich oligonucleotide compared to given reference CD spectra [60].

In terms of tertiary structure parameters (parallel, hybrid and antiparallel G4 structures), the deconvolution analysis performed on the CD spectra indicated that in the selected HEPES/Na^+^ buffer N.A. V7t1 and N.A. **bisV7t1TEG2D** almost exclusively formed parallel G4 structures, while additional conformations were evidenced for all the other investigated systems (Table 2). In detail, the CD spectra of N.A. **bisV7t1T7** and **bisV7t1HEG2** revealed that in both cases the parallel G4 conformation was prevalent (ca. 72 and 78%, respectively), accompanied by a small percentage of antiparallel G4 topology (ca. 20% in **bisV7t1T7**, ca. 23% in **bisV7t1HEG2**) and also a little amount of hybrid G4 conformations (ca. 7%) for **bisV7t1T7.**

In contrast, SVD analysis performed on the CD spectra of A. V7t1, **bisV7t1T7** and **bisV7t1HEG2** reflected the presence of a mixture of parallel and antiparallel G4 conformations, with prevalence of the latter ones (ca. 63–67%), and no contribution of hybrid G4 structures. Conversely, A. **bisV7t1TEG2D** preferentially adopted a parallel G4 (ca. 75%), accompanied by a ca. 25% hybrid G4.

Concerning the CD spectra recorded in the K^+^-rich medium, the deconvolution analysis showed that the CD spectra of N.A. V7t1 as well as **bisV7t1HEG2** and **bisV7t1TEG2D** in both N.A. and A. form were indicative of the exclusive presence in solution of parallel G4 structures. In turn, beside the prevalent parallel G4 conformation, additional G4 topologies were also predicted for the other samples (Table 2). Indeed, A. V7t1 showed both hybrid and antiparallel G4 structures in very small percentages (ca. 2 and 6%, respectively) and N.A. **bisV7t1T7** presented 4% of hybrid G4 conformations. Finally, A. **bisV7t1T7** showed parallel and hybrid-type G4 structures in comparable abundance (45 and 38%, respectively), along with a low percentage of antiparallel conformations (ca. 16%). Notably, these predictions are in agreement with the different spectral features observed for A. **bisV7t1T7**, particularly considering the CD signal maximum at 268 nm (Figure 3c, dashed green line) which can be explained with the co-existence of different G4 structures in different amounts (Table 2).

#### 2.4.3. CD Thermal Denaturation/Renaturation Measurements

The thermal stability of covalent V7t1 dimers was then analyzed by CD experiments, monitoring their melting and annealing profiles at the wavelength corresponding to the highest ellipticity observed.

**Figure 4:**
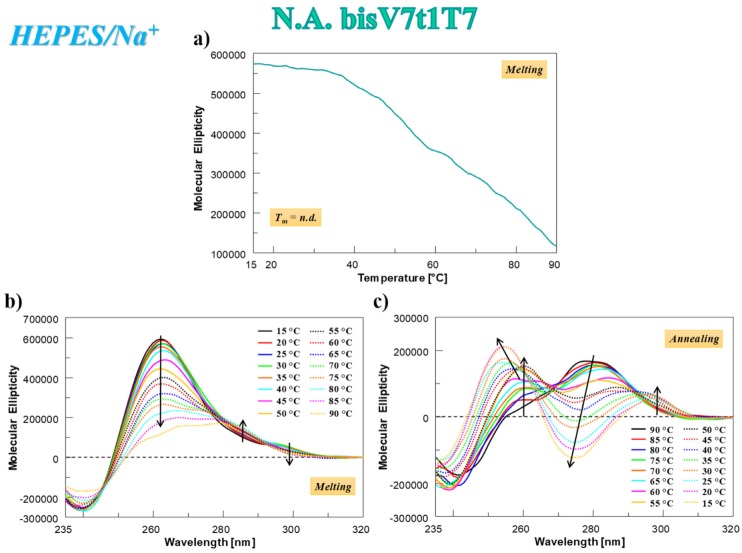
N.A. **bisV7t1T7** at 2 µM concentration in the selected HEPES/Na^+^ buffer: (**a**) CD-melting profile, recorded at 262 nm using a scan rate of 1 °C/min; (**b**,**c**) overlapped CD spectra recorded every 5 °C during the melting (**b**) and cooling processes (**c**). Arrows in panels b and c indicate the evolution of the CD signal over time. n.d. = not determined.

In the case of N.A. **bisV7t1T7** analyzed in HEPES/Na^+^ buffer solution, the CD-melting profile showed a marked decrease of the CD signal upon increasing the temperature (Figure 4a), not showing the typical sigmoidal behavior expected for a unique, cooperative transition, but rather a curve with at least two transitions, one below 50 °C and one at ca. 80 °C. This profile, resembling the one found for V7t1 [17], could be due to the presence of different species in solution (in accordance with the electrophoretic and chromatographic results) and/or to multi-process denaturation pathways. In addition, the CD-annealing profile—not superimposable to the corresponding heating profile—did not show a clearly defined sigmoidal behavior.

The analysis of the CD spectra of N.A. **bisV7t1T7** acquired every 5 °C during the melting and cooling processes revealed that its denaturation was not complete even at 90 °C (Figure 4b) and a marked reorganization of the G4 structure occurred during the cooling process (Figure 4c). In this thermal treatment, the initial band centered at 280 nm (Figure 4c, black line) decreased on varying the temperature and two positive bands (at 253 and 297 nm) appeared, indicating that not a single transition but multiple events occurred on decreasing the temperature. Remarkably, the initial CD spectral features of N.A. **bisV7t1T7** at 15 °C after the heating/cooling process were not restored (Figure 4b,c), as clearly evidenced by the direct comparison of the spectra before and after the thermal denaturation experiments in Figure S11a, indicating irreversible folding/unfolding processes. Indeed, in line with the SVD analysis results, an overall rearrangement of the preferred G4 conformations occurred, going from the mainly parallel G4 folding in the N.A. sample to a predominant contribution of the antiparallel G4 structuring in the A. **bisV7t1T7**.

As far as A. **bisV7t1T7** was concerned, both the CD-melting and annealing profiles did not show sigmoidal shapes, and the analysis of the spectra recorded on varying the temperature revealed a detectable rearrangement of its G4 structure in solution, evidenced by significant shifts of the maxima and minima of the main bands (Figure S11b,c).

Notably, in the case of A. **bisV7t1T7**, after heating/cooling experiments, a full recovery of the initial spectral features was observed (Figure S11d). Thus, although not showing a unique sigmoidal profile but rather a curve with multiple transitions, the thermal denaturation/renaturation processes of A. **bisV7t1T7** were overall reversible (Figure S11b–d). Interestingly, the spectrum recorded going back to 15 °C after the heating/cooling cycle at 1 °C/min (fast annealing, Figure S11e, blue line) was almost superimposable to that of the slowly A. **bisV7t1T7** (ca. 0.3 °C/min, Figure S11e, green line). Therefore, fast and slow annealing procedures gave comparable refolded G4 topologies, with similar contributions to the CD bands at 253/254 and 297 nm. (Figure S11e).

Quite similar was the behavior of **bisV7t1HEG2** analyzed in the same saline conditions. Indeed, CD-melting and annealing profiles showed non-sigmoidal curves, independently from the sample preparation procedure. The analysis of the CD spectra recorded on varying the temperature showed a general reduction of the band at 261 nm upon heating the N.A. sample (Figure S12a). Moreover, significant changes were found on cooling the N.A. sample (Figure S12b) as well as in both melting and annealing profiles of the A. sample (Figure S12c,d). In addition, for this sample, the spectra acquired during the CD thermal denaturation experiments denoted several shifts in the maxima and minima of the bands, overall indicating multiple events occurring on varying the temperature, plausibly related to the unfolding of different G4 species (Figure S12a–d).

The essentially parallel G4 structure present in N.A. **bisV7t1HEG2** consistently rearranged during the unfolding/folding processes, providing species with two positive bands at 254 and 297 nm (Figure 3b and Figure S11e), identified by the SVD analysis as mixtures of parallel and antiparallel G4 structures, with prevalence of the latter ones. The G4 structures of the A. sample were stable to further thermal treatments, completely recovering the initial spectral features (Figure S12f), also showing similar G4 foldings for both fast and slow annealing (Figure S12g, blue and green lines, respectively).

**Figure 5:**
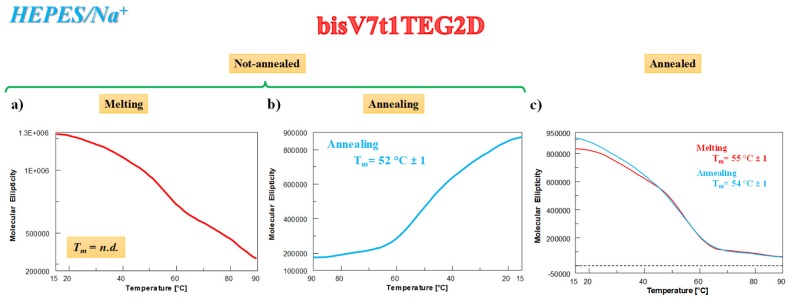
CD analysis on **bisV7t1TEG2D** at 2 µM concentration in the selected HEPES/Na^+^ buffer solution in both N.A. and A. form. (**a**) CD-melting and (**b**) CD-annealing profiles (red and light blue lines, respectively) of N.A. **bisV7t1TEG2D** recorded at 262 nm using a scan rate of 1 °C/min; (**c**) overlapped CD-melting and CD-annealing profiles (red and light blue lines, respectively) of A. **bisV7t1TEG2D**, both recorded at 262 nm (scan rate: 1 °C/min).

The behavior of **bisV7t1TEG2D** was significantly different from that observed for the other covalent V7t1 dimers in the HEPES/Na^+^ medium, resembling what already found during the UV-thermal experiments. Indeed, the CD-melting profile (Figure 5a) of the N.A. sample did not show a clearly defined sigmoidal behavior, while the CD-annealing profile provided a nice sigmoidal curve (Figure 5b), with apparent T_m_ values of 52 °C, indicative of stable G4 structures. The analysis of the CD spectra on varying the temperature showed an overall reduction of the main CD intensity band at 262 during the heating process (Figure S13a) and a detectable enhancing of the CD signal at 295 nm in cooling experiments (Figure S13b), consistently with the formation of alternative G4 conformations in solution (ca. 25% hybrid according to the SVD analysis).

Differently from N.A. **bisV7t1TEG2D**, the CD analysis of the A. sample showed nice sigmoidal denaturation pathways, with almost superimposable heating/cooling profiles and T_m_ values of 55/54 °C, respectively, indicating essentially reversible processes in the explored conditions (Figure 5c).

According to these results, the initial CD signal intensity of the N.A. **bisV7t1TEG2D** sample at 15 °C after the heating/cooling process was not completely restored (Figure S13c, green and blue lines, respectively), indicating irreversible folding/unfolding processes and further underlining the formation of G4 hybrid topologies in solution. These G4 structures, when subjected to the subsequent thermal treatments, almost completely recovered the initial spectral features (Figure S13d), with similar behavior for fast and slow annealing (Figure S13e, blue and green lines, respectively). The overall CD experiments results on **bisV7t1TEG2D** in both N.A. and A. form resemble those found in the UV-thermal measurements (Figure 2), also denoting in the Na^+^-rich medium a good accordance for the apparent T_m_ values derived by using the two techniques.

Monitoring the CD signal on varying the temperature in the selected TRIS/K^+^ buffer solution, in all cases the behavior of the samples was not dramatically affected by the annealing procedure, as already observed by PAGE and SE-HPLC studies. Indeed, the 263 nm-monitored CD-melting and annealing profiles of N.A. and A. V7t1 forms in the K^+^-rich buffer in no case showed a nice sigmoidal curve (as representatively reported for the N.A. sample in Figure S14a), but at least two barely hinted transitions, quite similar to those observed in the previously investigated Na^+^-rich medium [17].

In contrast, the CD melting experiments, monitored for **bisV7t1T7** (Figure S14b,c) and **bisV7t1HEG2** (Figure S14d,e) at their ellipticity maxima, provided in all cases a nice sigmoidal profile, featured by some hysteresis. Particularly, apparent T_m_ values in the range 53–64 °C were found, indicating the formation of quite stable G4 structures.

Slight differences were observed for the CD-thermal experiments performed on **bisV7t1TEG2D** in the selected TRIS/K^+^ buffer solution (Figure S14f,g), essentially resembling the behavior already observed in the Na^+^-rich medium (Figure 2 and Figure 5). Indeed, the CD-melting profile of the N.A. sample did not show the characteristic sigmoidal shape of a unique, cooperative transition (Figure S14f, red line), while the CD-annealing experiment provided a nice sigmoidal behavior with apparent T_m_ value of 56 °C (Figure S14f, orange line). For the A. **bisV7t1TEG2D** sample, both heating and cooling experiments gave sigmoidal profiles, with apparent T_m_ values of 60 and 54 °C, respectively (Figure S14g, red and orange lines), indicating some hysteresis under the experimental conditions tested.

In Table S1, an overview of the apparent T_m_ values derived by CD-monitored thermal denaturation experiments is reported.

### 2.5. Binding Experiments with VEGF_165_ Protein

VEGF-binding properties of covalent V7t1 dimers were studied by EMSA experiments under non-denaturing conditions, which is a sensitive and widely used method to detect protein-nucleic acid interactions [61]. In fact, the electrophoretic mobility of an oligonucleotide is typically high when free in solution, and significantly retarded when complexed with its target protein [17,20,61]. In parallel experiments, both N.A. and A. samples were incubated at 4 °C for 30 min with 1.3 eq. of VEGF_165_ and the resulting mixtures analyzed by 7% native PAGE using the free oligonucleotides as controls. The gels were subjected to a double staining procedure, using first a staining specific for nucleic acids (GelGreen) and then one specific for proteins (Coomassie) [17].

**Figure 6:**
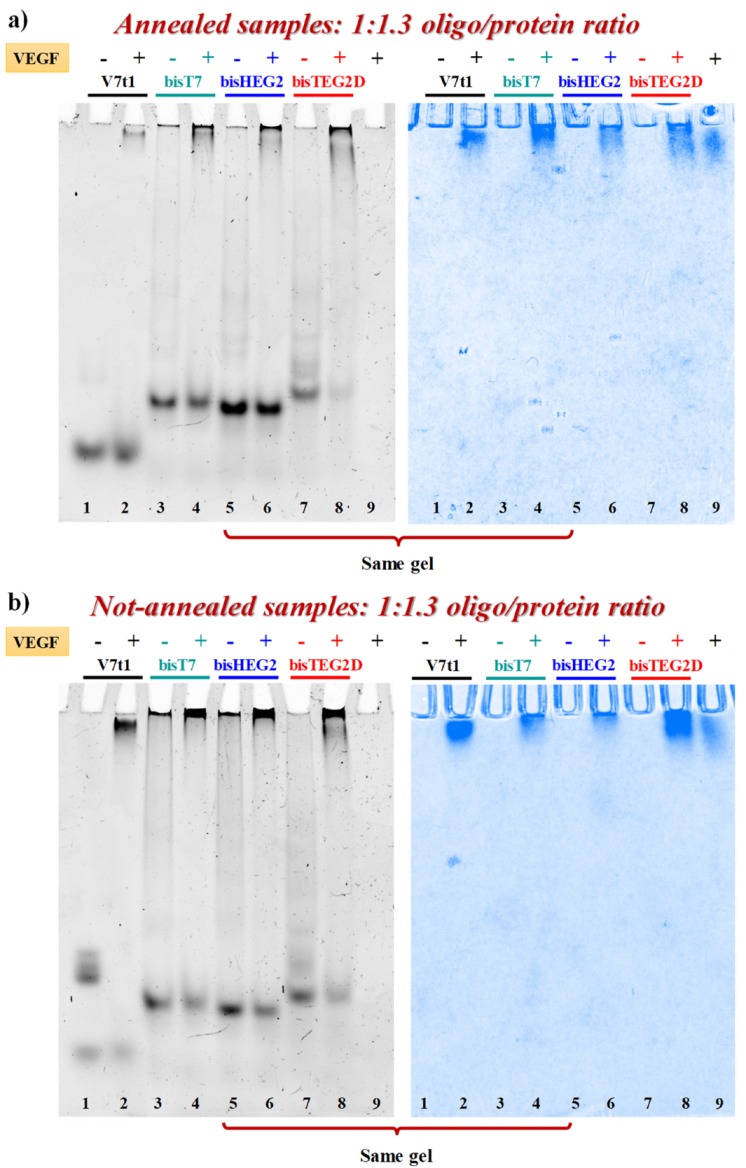
Native 7% electrophoretic mobility shift assay (EMSA) of A. (**a**) and N.A. (**b**) V7t1 and covalent V7t1 dimers incubated in the presence (+) or absence (**−**) of VEGF_165_. GelGreen- and Coomassie-stained gels (left and right, respectively). 30 pmol of each aptamer were incubated with 40 pmol of the protein in a final volume of 9 μL in the selected HEPES/Na^+^ buffer, thus obtaining a final 1:1.3 oligo/protein ratio. Gels were run at constant 45 V for 2.3 h at r.t. in TAE 1X buffer.

When each A. sample was mixed with the protein, nucleic acid staining revealed the appearance of a new retarded band, attributable to the formation of the aptamer-protein complex. However, the bands corresponding to **bisV7t1T7** and **bisV7t1HEG2** did not completely disappear (Figure 6a, lanes 4 and 6, left) while A. **bisV7t1TEG2D** (Figure 6a, lane 8, left) almost fully bound the protein in the same conditions. Additionally, Coomassie staining confirmed these findings, showing a sharper migration profile for VEGF_165_ when bound to the aptamer, compared to the smeared migration occurring when the protein was free in solution, as previously reported (Figure 6a, right) [17].

The same experiments were also performed on the N.A. samples (Figure 6b, left) but in these cases the presence of large aggregates, especially for **bisV7t1T7** and **bisV7t1HEG2**, complicated the results interpretation. Indeed, the bands corresponding to the covalent dimers, and particularly of **bisV7t1TEG2D**, were detectably reduced when the oligonucleotides were mixed with the protein, thus confirming the binding, even if in no case they completely disappeared.

The EMSA results suggested **bisV7t1TEG2D** as the oligonucleotide with the highest affinity for VEGF_165_ among the different covalent V7t1 dimers here investigated in both N.A. and A. form. These data could be likely associated to the different linker used to connect the two V7t1 sequences in **bisV7t1TEG2D**, providing an inversion of polarity which can probably promote a different three-dimensional folding of this V7t1 dimer, preferred by the protein.

In order to confirm the binding specificity of V7t1 and its covalent dimers for the VEGF_165_ protein, we also investigated possible unspecific binding using as control bovine serum albumin (BSA). Thus, in parallel experiments, both N.A. and A. samples—dissolved in the selected HEPES/Na^+^ buffer—were incubated with BSA following the same protocol exploited for VEGF_165_ binding and then the aptamer/protein mixtures (in 1:1.3 ratio) were analyzed by EMSA assays, including the free oligonucleotides as well as pure BSA as negative controls (Figure S15). After nucleic acid staining, the gel showed that the bands of both A. and N.A. oligonucleotides treated with BSA (Figure S15a,b, respectively, left) had the same mobility as the free aptamers. The absence of differences in the migration ability and/or in the intensity of the V7t1 and its covalent dimers bands unambiguously demonstrated that no binding occurred between these oligonucleotides and BSA, as also previously evidenced for V7t1 even if under different conditions [62]. In turn, Coomassie staining (Figure S15a,b, right) did not reveal any difference in the migration ability of the protein bands depending on whether alone in solution or incubated with the aptamers, further confirming the absence of unspecific binding between the selected DNA sequences and BSA. Therefore, our EMSA results overall proved that VEGF_165_ binding of V7t1 and its dimers was due to specific target recognition.

### 2.6. In vitro Bioactivity Studies

Following conformational behavior and molecularity investigations, targeted in vitro bioscreens were set up in order to evaluate the activity of V7t1 and its covalent V7t1 dimers on human breast adenocarcinoma MCF-7 cells in terms of antiproliferative effects. These breast cancer cells were specifically selected as a relatively V7t1-sensitive cell line, already proved to be responsive to this oligonucleotide, even if only in the presence of specific transfecting agents [15].

**Figure 7:**
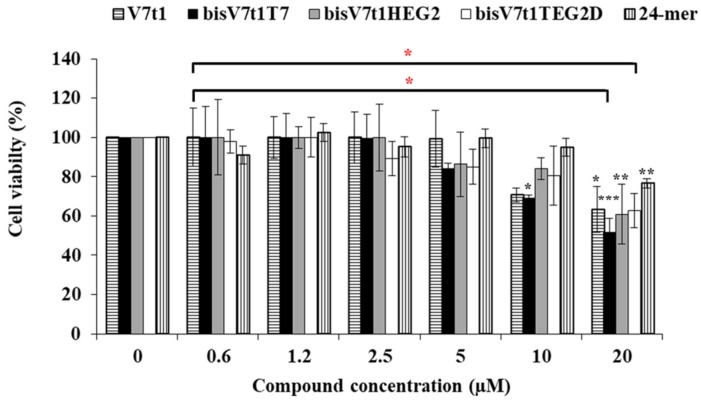
Cell viability assay of V7t1, **bisV7t1T7**, **bisV7t1HEG2**, **bisV7t1TEG2D** and a control 24-mer on MCF-7 cells tested by MTT assays. Cell viability is reported as the % of live cells with respect to control untreated cells (100% cell viability). Reported results derive from 4 independent experiments. Statistical analysis was performed by using Student’s t-Test by comparing all samples to the control cells (* *P* < 0.05, ** *P* < 0.01 or *** *P* < 0.001) and ANOVA test by comparing all covalent V7t1 dimers to V7t1 (* *P* < 0.05).

The cytotoxic activity induced by the here studied oligonucleotides was assessed analyzing a range of concentrations, from 0.6 to 20 μM, using the standard tetrazolium-based (MTT) assay after 48 h of treatment. The MTT assay results showed a slight cytotoxicity of all the covalent V7t1 dimers starting from 5 µM concentration, producing a ca. 20% cell viability reduction. At the highest concentration tested (20 µM), enhanced antiproliferative effects for all these compounds were found, in line with the bioactivity increase of V7t1, thus demonstrating a dose-dependent effect (Figure 7).

We also tested a single-stranded oligonucleotide, selected as a negative control, i.e., the 24-mer (^5′^TCACACACACACACACACACACTT^3′^) non-containing guanines and representing a useful DNA model system [63,64]. This single-stranded 24-mer showed a modest toxicity (ca. 20% cell viability reduction) only at the highest concentration tested (20 μM), proving not to be significant (ANOVA test analysis) with respect to V7t1 and its dimeric analogues (Figure 7).

The half maximal inhibitory concentration (IC_50_) is the concentration of a drug required to obtain 50% inhibition of cell proliferation in vitro and measures the effectiveness of a substance in inhibiting a specific biological function. All the analyzed compounds displayed IC_50_ values slightly higher than 20 µM, except **bisV7t1T7**, for which an IC_50_ value of 19 µM was obtained from the experimental results (Table 3), indicative of a good cytotoxic effect on these human cancer cells. In the case of the control non G4-forming oligonucleotide, the extrapolated IC_50_ value was found to be 50 µM, indicative of a significantly lower cytotoxicity with respect to V7t1 aptamer or its dimers. The overall effect of these dimeric aptamers on cell viability could be rationalized considering the ability of these molecules to perturb the VEGF/Notch signaling pathways, as also previously found for other VEGF-targeting aptamers [65]. Indeed, interference in these biological processes can eventually cause tumor cell inhibition, since the Notch signaling pathway is involved in cell-cell communication and cell differentiation [21,66].

## 3. Conclusions

Several bivalent or even multivalent aptamers have been recently proposed for different biologically relevant targets (proteins and cellular receptors) with the aim to identify better performing analogues with enhanced affinity or activity. A dimeric structure represents the simplest multivalent system and nucleic acid aptamers are especially suitable for the design of these constructs as they can be easily and ad hoc chemically modified.

Starting from our recent and intriguing findings on the ability of V7t1 to form a dimeric G4 structure with preferential binding capability toward VEGF_165_ compared to the monomeric V7t1 species, in this study we selected a focused set of covalent V7t1 dimers in order to identify novel anti-VEGF aptamers with improved affinity and/or bioactivity. In our expectations, the covalent connection of two V7t1 sequences via suitable linkers should somehow pre-organize its G4 structures, in principle reducing the structural polymorphism of V7t1 and enhancing its effective bioactive form.

In detail, three covalent V7t1 dimers, here named **bisV7t1T7**, **bisV7t1HEG2** and **bisV7t1TEG2D**, were ad hoc designed with linkers connecting the V7t1 sequences of similar overall lengths, but different chemical nature. In particular, **bisV7t1T7** and **bisV7t1HEG2** presented two tandem V7t1 sequences, both in the 5′→3′ direction, connected respectively by a T_7_ and a HEG-based linker. In turn, in **bisV7t1TEG2D**, a TEG-based spacer joined the 3′ extremities of two V7t1 25-mers providing the final dimer with a 3′-3′ inversion of polarity.

Different biophysical techniques were used to elucidate the conformational behavior, thermal stability and molecularity of the G4 species formed in two different solutions (a Na^+^-rich and a K^+^-rich buffer, indicated respectively as HEPES/Na^+^ and TRIS/K^+^), with and without prior annealing.

Gel electrophoresis and SE-HPLC results were in accordance, showing that in HEPES/Na^+^ buffer the number and nature of the species formed in solution were dramatically influenced by the sample preparation procedure. Indeed, N.A. samples essentially formed species with the mobility and size expected for covalent V7t1 dimers, but were also accompanied by large aggregates, which in contrast were sensibly reduced after annealing the samples. On the contrary, the investigation in TRIS/K^+^ buffer revealed no detectable difference between the N.A. and A. samples, showing in all cases the presence of the expected species along with higher order aggregates.

The UV and CD studies reflected this general picture, showing CD spectra and CD- as well as UV-melting curves similar for the N.A. and A. samples in TRIS/K^+^ buffer, and remarkably different spectral features in HEPES/Na^+^ buffer depending if the samples were annealed or not. CD-thermal denaturation/renaturation experiments performed in the Na^+^-rich buffer revealed in all cases, except for **bisV7t1TEG2D**, complex heating/cooling pathways, evidencing at least two main transitions in solution. Conversely, all the here investigated covalent V7t1 dimers showed in TRIS/K^+^ buffer a nice sigmoidal behavior, corresponding to species with high T_m_ values, indicative of stable G4 structures.

As far as the evaluation of the VEGF_165_ binding properties was concerned, **bisV7t1TEG2D** showed enhanced affinity for the protein in both N.A. and A. form compared to **bisV7t1T7** and **bisV7t1HEG2**, even if slightly reduced with respect to V7t1. These results could be likely associated to the different linker exploited to connect the V7t1 sequences in **bisV7t1TEG2D**, providing for this aptamer an inversion of polarity which can promote a different 3D folding, preferred by the protein. Indeed, this would point to the parallel G4 folding as the one better recognized by VEGF_165_, considering the large preference of **bisV7t1TEG2D** for this G4 conformation.

On the other hand, the evaluation of the antiproliferative activity on human adenocarcinoma breast MCF-7 cancer cells did not show a sensibly different bioactivity in the investigated compounds, in all cases proving to be essentially similar to the one of monomeric V7t1, with IC_50_ values around or slightly higher than 20 µM, and more marked than that of a control oligonucleotide.

In conclusion, a detailed characterization of novel covalent V7t1 dimers has been here carried out, in the frame of a project aimed at developing anticancer strategies based on VEGF targeting.

The enhancement in affinity and/or activity which can be achieved with the use of dimeric aptamer-based constructs critically depends on the length and chemical nature of the connecting linker joining the two oligonucleotide sequences, as this determines their presentation to the target protein. Unfortunately, there are no general rules for the construction of effective dimeric aptamers and typically a trial-and-error approach, in a successive refinement work, is pursued. This goal is more and more challenging when, as in our case, detailed structural data of the target protein or of the aptamer/protein binding sites are not available. Thus, even if the dimerization approach is often the strategy of choice to improve the general properties of aptamers, the here described dimeric variants of V7t1—in analogy with many other literature cases—did not lead to substantial affinity improvement toward the selected protein target. However, the preliminary biological experiments indicated the covalent V7t1 dimers as promising cytotoxic compounds.

Among the analogues here investigated, particularly interesting proved to be **bisV7t1TEG2D**, incorporating a non-nucleotide linker introducing a 3′-3′ inversion of polarity site in the middle of its chain, which proved to favor the parallel G4 conformation, reducing the overall polymorphism of V7t1. Based on the large amount of data here acquired, it will be possible to further improve its connecting linker properties, ad hoc varying its overall flexibility or length. All these modifications can be easily synthetically obtained and will be extended also to other G4-forming aptamers to produce a new generation of dimeric aptamers with enhanced functionalities.

## 4. Materials and Methods

### 4.1. General Methods

All the reagents and solvents were of the highest commercially available quality and were used as received. Acrylamide, GelGreen Nucleic Acid Stain, Bromophenol blue (BPB), Gel Loading Buffer 4X, 6X Orange DNA Loading Dye and Tris-Borate-EDTA (TBE) 10X were purchased from VWR. Ammonium persulfate (APS) and tetramethylethylenediamine (TEMED) were purchased from Sigma Aldrich. All the oligonucleotides here studied (Table 1) were purchased from biomers.net GmbH (Ulm, Germany), as HPLC-purified oligomers:

▪ V7t1 (^5′^TGTGGGGGTGGACGGGCCGGGTAGA^3′^);
▪ bisV7t1T7 (^5′^V7t1^3′^TTTTTTT^5′^V7t1^3′^);
▪ bisV7t1HEG2 (^5′^V7t1^3′^C_24_H_51_O_20_P_3_^5′^V7t1^3′^);
▪ bisV7t1TEG2D (^5′^V7t1^3′^C_25_H_53_N_2_O_24_P_5_^3′^V7t1^5′^).

Evidence of the oligonucleotide identity and purity was obtained by MALDI-TOF mass spectrometry and HPLC data, provided by the commercial suppliers. The purity of these oligonucleotides was further confirmed by denaturing 20% PAGE analysis.

The 24-mer sequence (^5′^TCACACACACACACACACACACTT^3′^), used as negative oligonucleotide control in the MTT assays, was obtained as reported in previous works [63,64].

Recombinant human VEGF_165_ (GenScript) was purchased from TwinHelix srl (Milan, Italy) and prepared according to the manufacturer’s instructions.

Bovine serum albumin (BSA) was purchased from Thermo Scientific™ (Waltham, MA, USA).

### 4.2. Preparation of the Oligonucleotide Samples

Lyophilized V7t1 and covalent V7t1 dimers (here named as **bisV7t1T7**, **bisV7t1HEG2** and **bisV7t1TEG2D**, Table 1) were dissolved in a defined volume of Milli-Q water. Their concentrations were determined by UV measurements on a JASCO V-530 UV-vis spectrophotometer equipped with a Peltier Thermostat JASCO ETC-505T, using a 1 cm path length cuvette (1 mL internal volume, Hellma), recording the absorbance at 260 nm and 90 °C. The following molar extinction coefficients were used: ε_260_ = 273.522 M^−1^·cm^−1^ for V7t1, 619.962 M^−1^·cm^−1^ for **bisV7t1T7** and 547.044 M^−1^·cm^−1^ for both **bisV7t1HEG2** and **bisV7t1TEG2D**, as calculated for the unstacked oligonucleotides, assuming that the inserted non-nucleotide linkers did not provide any significant contribution to the UV absorbance at 260 nm. The UV spectra were recorded in the range 200–320 nm with a medium response, a scanning speed of 100 nm/min and a 2.0 nm bandwidth with the appropriate baseline subtracted. Taking a suitable aliquot from the initial stock solutions in H_2_O, all the investigated oligonucleotides were then diluted in the selected Na^+^- (25 mM HEPES, 150 mM NaCl, pH = 7.4, here indicated as HEPES/Na^+^) or K^+^-rich (10 mM Tris, 100 mM KCl, pH = 7.1, here indicated as TRIS/K^+^) buffer. In particular, the not-annealed (N.A.) samples were prepared by simple dilution in the selected buffer at r.t. from a concentrated stock solution in pure water. In contrast, the annealed (A.) samples were obtained by heating the appropriate aptamer solution at 100 °C for 5 min and then allowing it to slowly cool to r.t. overnight, to allow their structuring into the thermodynamically most stable conformations [67]. N.A. and A. samples were then kept at 4° C until subsequent use.

### 4.3. Gel Electrophoresis Analysis

***Denaturing PAGE.*** A total of 20 pmol of V7t1 and V7t1 dimers in water were mixed with formamide (1:2, *v*/*v*), heated at 95 °C for 5 min, then left in contact with ice until subsequent loading. Thereafter, all the samples—supplemented with 6X Orange DNA Loading Dye immediately before loading—were analyzed by electrophoresis on 20% denaturing polyacrylamide gels using 8 M urea in TBE (Tris-Borate-EDTA) 1X as running buffer. The gels were run at r.t., at constant 200 V for 3.5 h, then stained with GelGreen Nucleic Acid Stain for 30 min and finally visualized with a UV transilluminator (ChemiDoc XRS, BioRad, Milan, Italy). The experiment was performed in triplicate.

***Native PAGE.*** Not-annealed and slowly annealed samples of V7t1 and its covalent dimers, dissolved at 4 μM concentration in both the selected HEPES/Na^+^ or TRIS/K^+^ buffer solutions, were loaded on 10% polyacrylamide gels in TBE 1X as running buffer. All the samples were supplemented with BPB Gel Loading Buffer 4X immediately before loading and then run, under native conditions, at constant 70 V at r.t. for 1.75 or 2 h, as specified. Gels were stained with a GelGreen solution (supplemented with NaCl 0.1 M) for 30 min and finally visualized with a UV transilluminator. Each experiment was performed at least in triplicate.

***Native agarose gel electrophoresis.*** Agarose solution in TBE 1X (2% *w*/*v*) was mixed with 0.01 % (*v*/*v*) of GelGreen stock reagent before agarose solidification (pre-cast protocol). Not-annealed and slowly annealed samples of V7t1 and covalent V7t1 dimers were dissolved at 4 μM concentration in both the selected HEPES/Na^+^ or TRIS/K^+^ buffer solutions as well as in the amine-free buffers, i.e., 150 mM NaCl (pH = 7.4), as Na^+^-rich buffer and 100 mM KCl (pH = 7.3), as K^+^-rich buffer.

All the samples were supplemented with glycerol (5% *w/v* in the final solution) immediately before loading them on the gel and then run, under native conditions, at constant 60 V at r.t. for 2 h in TBE 1X as running buffer. The gel was then visualized with a UV transilluminator. Each experiment was performed at least in triplicate.

### 4.4. Size Exclusion Chromatography

SE-HPLC analyses were performed using an Agilent HPLC system, equipped with a UV/vis detector, on a Yarra 3 µm analytical column (300 × 4.60 mm; Phenomenex). The elution was monitored at λ = 254 nm with 0.35 mL·min^−1^ flow rate. The mobile phases consisted of HEPES/Na^+^ (25 mM HEPES, 150 mM NaCl, pH = 6.8) or TRIS/K^+^ (10 mM Tris, 100 mM KCl, pH = 7.0) buffer solutions. Both N.A. and A. V7t1 and the covalent V7t1 dimers were investigated at 2 µM concentration in the selected buffer solutions, using V7t1 as control. Each experiment was performed in triplicate. The error associated with the retention time (t*_R_*) determination is within ± 5%.

### 4.5. UV Spectroscopy

The UV spectra measurements were performed on a JASCO V-630 UV-vis spectrophotometer equipped with a Peltier Thermostat JASCO ETCS-761, using 1 cm path length cuvette (1 mL internal volume, Hellma). All the covalent V7t1 dimers were dissolved in the selected buffer solution, so to obtain 2 μM solutions, and analyzed as such or in the annealed form. The thermal difference spectra (TDS) were obtained by subtracting the UV spectrum recorded at a temperature below the T_m_ (15 °C), at which the aptamer is fully structured, from that obtained at a temperature above the T_m_ (90 °C), when the oligonucleotide is fully destructured [49,50,51]. In detail, the UV-vis spectra were recorded in the 220–320 nm range using a scanning speed of 100 nm/min and the appropriate baseline subtracted, as previously described [42,50].

The absorbance vs. temperature profiles of N.A. and A. covalent V7t1 dimers were recorded following the absorbance changes (at 295 or 260 nm, as specified) in the temperature range 15–90 °C [50,68]. The T_m_ values, where possible, were calculated as the maxima of the first derivative plots of the melting curves (associated error: ±1 °C). Each experiment was performed in duplicate.

### 4.6. Circular Dichroism (CD) Spectroscopy

CD spectra and CD-monitored melting/annealing curves were recorded on a Jasco J-715 spectropolarimeter equipped with a PTC-348WI Peltier-type temperature control system (JASCO Europe Srl, Cremella (LC), Italy), using a quartz cuvette with a path length of 1 cm (3 mL internal volume, Hellma). CD parameters for spectra recording were the following: spectral window of 235-320 for HEPES/Na^+^ and 220–320 nm for TRIS/K^+^, data pitch 1 nm, band width 2 nm, response 4 s, scanning speed 100 nm/min, 3 accumulations, with the appropriate subtraction of the background scan with proper blank [59].

All the oligonucleotides were analyzed at 2 μM concentration in the selected buffer solutions. Thermal denaturation/renaturation curves were recorded following the CD signal—at the maximum ellipticity observed for each oligonucleotide system—vs. the temperature (scan rate of 1.0 °C/min) and recording spectra in 5 °C steps, in the 15–90 and 15–95 °C temperature range, respectively for HEPES/Na^+^ and TRIS/K^+^ buffer solutions. Each experiment was performed in duplicate. The molar ellipticity (θ) (deg·cm^2^·dmol^−1^) was calculated from the equation (θ) = θ_obs_/10 × *l* × *C*, where θ_obs_ is the observed ellipticity (mdeg), *C* is the oligonucleotide molar concentration and *l* is the optical path length of the cell (cm). The T_m_ values were estimated as the maxima of the first derivative plots of the melting/annealing curves and the error associated with the T_m_ determination was ± 1 °C.

For the singular value decomposition (SVD) analysis, CD spectra were also normalized to molar circular dichroism, Δε (M^−1^·cm^−1^) = θ/(32980 × c × l) based on G4 strand concentration, where θ is the CD ellipticity in millidegrees, c is DNA concentration in mol·L^−1^ and l is the path length in cm. Then, the obtained spectra were analyzed by the software developed by del Villar-Guerra et al. [60].

### 4.7. Electrophoresis Mobility Shift Assay (EMSA)

For EMSA experiments, 30 pmol of each oligonucleotide in both N.A. and A. form were incubated with 40 pmol of VEGF_165_ or BSA (1:1.3 oligo/protein ratio). All the oligonucleotide + protein samples were incubated in HEPES/Na^+^ buffer for 30 min at 4 °C. Glycerol was added to all the samples to a final concentration of 5% immediately before loading on the gel. Electrophoresis was performed on 7% polyacrylamide gels in TAE (Tris Acetate EDTA) 1X, pH = 7.8, at constant 45 V for 2.3 h [17,61]. Gels were then stained for 30 min with GelGreen Nucleic Acid Stain and visualized on a UV transilluminator (BioRad ChemiDoc XRS). After the DNA staining, the gels were washed in water and stained again with Colloidal Coomassie G-250 to visualize the protein position [17].

### 4.8. In vitro Biological Evaluation

***Eukaryotic cell cultures and cell viability MTT assays.*** Human breast adenocarcinoma MCF-7 cells were cultured in high-glucose Dulbecco’s modified Eagle’s medium (DMEM) supplemented with 10% fetal bovine serum (FBS), 1% penicillin-streptomycin at 37 °C in the presence of 5% carbon dioxide (CO_2_). MTT cell viability assays were performed upon seeding MCF-7 cells on 96-well plates at a density of 3 × 10^3^ cells/well in 0.1 mL of complete DMEM. After 24 h, the cells were incubated in the presence of increasing concentrations (0.6–20 μM) of samples and incubated for 48 h. Cell viability was evaluated by adding the MTT reagent (3-(4,5-dimethylthiazol-2-yl)-2,5-diphenyltetrazolium bromide) diluted at 0.5 mg/mL in DMEM without red phenol (0.1 mL/well). After 4 h of incubation at 37 °C, the resulting insoluble formazan salts were solubilized in 0.01 N HCl in anhydrous isopropanol and quantified by measuring the absorbance at 570 nm, using an automatic plate reader spectrophotometer (GloMax^®^ Discover System, Promega, Madison, Wisconsin, USA). Cell survival was expressed as percentage of viable cells in the presence of the aptamers, with respect to control cells grown in their absence. In all the experiments, controls were performed by supplementing cell cultures with identical volumes of the sole buffer for the same incubation time. Data were obtained from at least three independent experiments.

***Statistical analysis.*** Results are presented as the mean ± standard deviation (SD) of at least three independent experiments. Statistical significance was assessed by using either Student’s t-Test or ANOVA test. All samples were compared to the control (in the absence of treatment) and to V7t1-tretaed cells; significant differences were indicated as * *P* < 0.05, ** *P* < 0.01 or *** *P* < 0.001 in the case of Student’s t-Test and as * *P* < 0.05 in the case of ANOVA test.

## Figures and Tables

**Table 1:** Molecular structure of the covalent V7t1 dimers investigated in this study.

Name	Sequence	Linker Length
**bisV7t1T7**	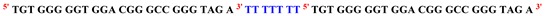	**42**
**bisV7t1HEG2**	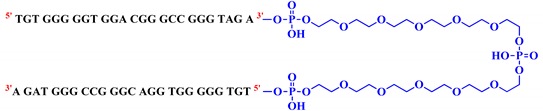	**40**
**bisV7t1TEG2D**	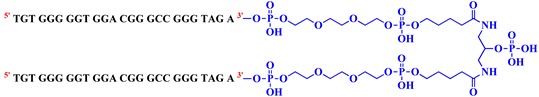	**40**

The sub-structure of the linkers exploited to connect the two V7t1 sequences is highlighted in blue. The linker length is expressed in number of chemical bonds present in the linker joining the phosphate extremities of the V7t1 sequences (starting from the terminal P atoms).

**Table 2:** Prediction of relative abundance of the G4 topologies adopted by N.A. and A. samples.

Prediction of G4 Topologies Relative Abundance from SVD Analysis of CD Spectra			
* **HEPES/Na^+^** *			
	**Parallel (%)**	**Hybrid (%)**	**Antiparallel (%)**
**N.A. V7t1**	100	1	0
**A. V7t1**	36	0	63
**N.A. bisV7t1T7**	72	7	20
**A. bisV7t1T7**	32	0	67
**N.A. bisV7t1HEG2**	78	0	23
**A. bisV7t1HEG2**	34	0	65
**N.A. bisV7t1TEG2D**	100	1	0
**A. bisV7t1TEG2D**	75	26	0
* **TRIS/K^+^** *			
**N.A. V7t1**	100	1	0
**A. V7t1**	92	2	6
**N.A. bisV7t1T7**	97	4	0
**A. bisV7t1T7**	45	38	16
**N.A. bisV7t1HEG2**	100	1	0
**A. bisV7t1HEG2**	100	1	0
**N.A. bisV7t1TEG2D**	100	1	0
**A. bisV7t1TEG2D**	100	1	0

The prediction for V7t1 and its covalent dimers was obtained by singular value decomposition (SVD) analysis of the CD spectra recorded in both the selected HEPES/Na^+^ and TRIS/K^+^ buffer solutions, performed by exploiting the software developed by del Villar-Guerra et al. [60]. Deviations from 100% (±1%) are due to significant digits approximation of the values originally obtained by the simulations.

**Table 3:** IC_50_ values of V7t1, its covalent dimers and a control 24-mer on MCF-7 cells after 48 h treatment.

Molecule	IC_50_ (µM)
V7t1	>20 µM
bisV7t1T7	19 µM
bisV7t1HEG2	>20 µM
bisV7t1TEG2D	>20 µM
**control 24-mer**	>20 µM

## Supplementary Materials

Supplementary materials can be found at https://www.mdpi.com/1422-0067/21/6/1963/s1.
