# Supplementary material for: Tuning the Polymorphism of the Anti-VEGF G-rich V7t1 Aptamer by Covalent Dimeric Constructs
Source: Int J Mol Sci. 2020 Mar 13;21(6):1963. doi: 10.3390/ijms21061963 (PMC7139925; doi:10.3390/ijms21061963)
Supplement: Supplementary file 1 [file ijms-21-01963-s001.pdf]

Supplementary Material

Tuning the Polymorphism of the Anti-VEGF G-rich V7t1 Aptamer by Covalent Dimeric Constructs

Claudia Riccardi [1], Domenica Musumeci [1,2], Chiara Platella [1], Rosa Gaglione [1], Angela Arciello [1,3] and Daniela Montesarchio [1,*]

[1] Department of Chemical Sciences, University of Naples Federico II, Via Cintia 21, I-80126, Napoli, Italy; claudia.riccardi@unina.it (C.R.); domenica.musumeci@unina.it (D.Mu.); chiara.platella@unina.it (C.P.); rosa.gaglione@unina.it (R.G.); angela.arciello@unina.it (A.A.)

[2] Institute of Biostructures and Bioimages (IBB), CNR, Via Mezzocannone 16, I-80134 Napoli, Italy

[3] National Institute of Biostructures and Biosystems (INBB), 00136 Rome, Italy

* Correspondence: daniela.montesarchio@unina.it

Table of contents

Figure S1. Schematic representation of the sequences of the covalent V7t1 dimers **pag. S3**

Figure S2. Molecular structure of the functionalized CPG-based solid supports and linker building blocks used for the oligonucleotide synthesis **pag. S4**

Figure S3. 20 % polyacrylamide denaturing gel electrophoresis analysis **pag. S5**

Figure S4. 10 % polyacrylamide native gel electrophoresis analysis **pag. S6**

Figure S5. 2 % agarose native gel analysis in amine-free buffers **pag. S7**

Figure S6. Size exclusion HPLC analysis in HEPES/Na^+ buffer solution **pag. S8**

Figure S7. Size exclusion HPLC analysis in TRIS/K^+ buffer solution **pag. S9**

Figure S8. TDS profiles in HEPES/Na^+ buffer solution **pag. S10**

Figure S9. TDS profiles in TRIS/K^+ buffer solution **pag. S11**

Figure S10. UV analysis on **bisV7t1T7** at 260 nm **pag. S12**

Figure S11. CD analysis on **bisV7t1T7** in HEPES/Na^+ buffer solution **pag. S13**

Figure S12. CD analysis on **bisV7t1HEG2** in HEPES/Na^+ buffer solution **pag. S14**

Figure S13. CD analysis on **bisV7t1TEG2D** in HEPES/Na^+ buffer solution **pag. S15**

Figure S14. CD analysis in the selected TRIS/K^+ buffer solution **pag. S16**

Table S1. T_m values obtained by CD-monitored thermal denaturation experiments **pag. S17**

Figure S15. EMSA experiments with BSA **pag. S18**

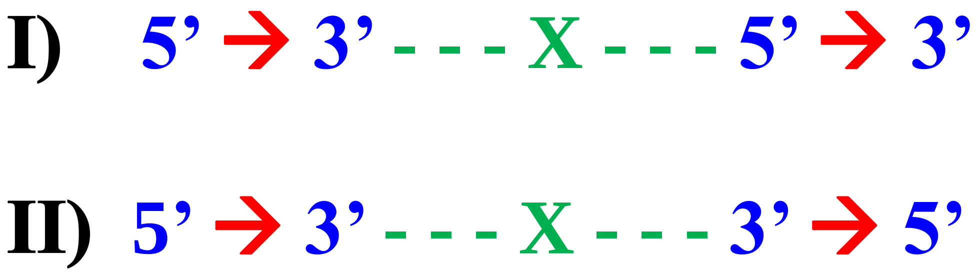

Figure S1. Polarity of the two strands in the V7t1 tandem sequences linked by a generic linker, indicated with ---X---. Scheme (**I**) represents the overall structure present in **bisV7t1T7** and **bisV7t1HEG2** in which both V7t1 strands have the 5′→3′ direction, while Scheme (**II**) was exploited in **bisV7t1TEG2D** with an inversion of polarity site.

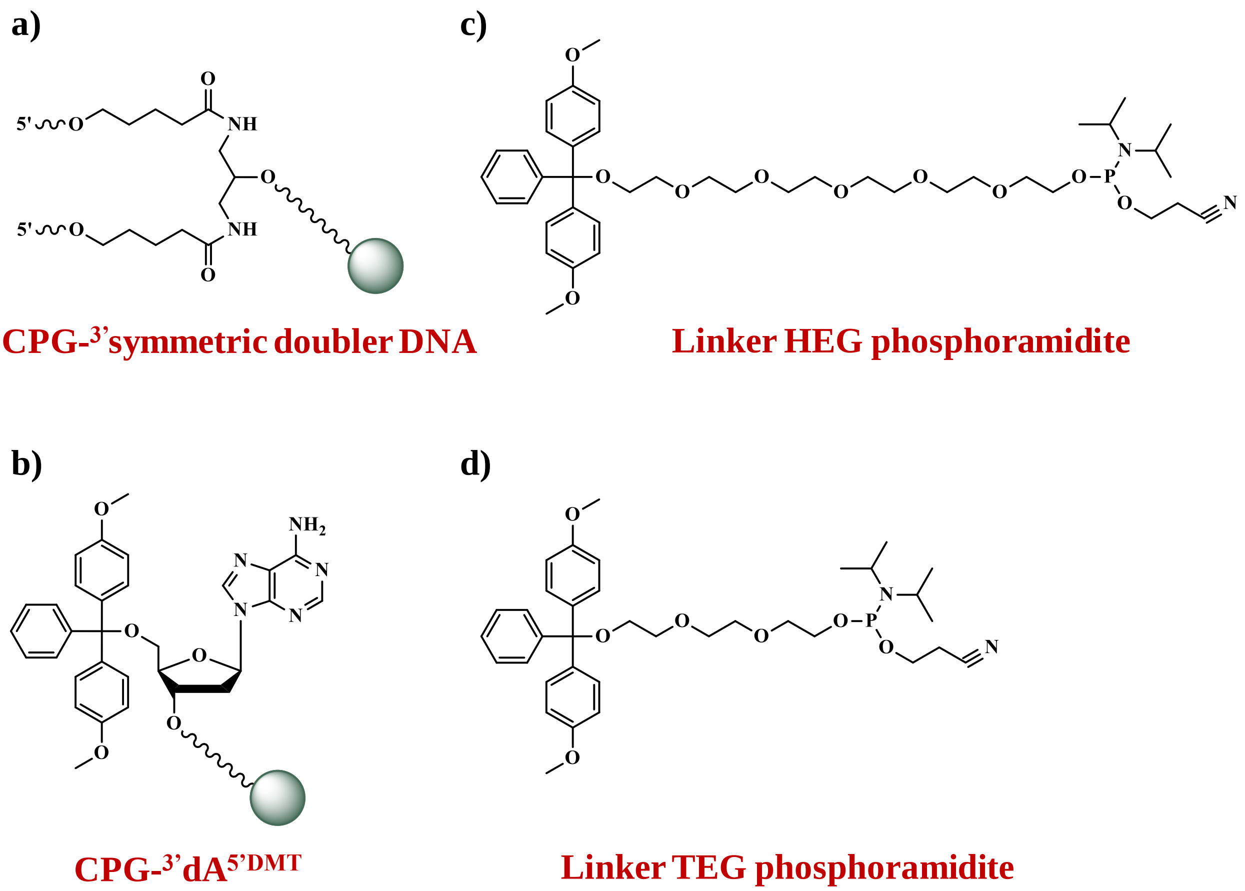

Figure S2. Molecular structure of the functionalized CPG-based solid supports and linker building blocks used for the oligonucleotide synthesis: (**a**) CPG-^{3}symmetric doubler DNA solid support for **bisV7t1TEG2D**; (**b**) CPG-$^{3'}$dA$^{5'DMT}$ solid support for **bisV7t1T7** and **bisV7t1HEG2**; (**c**) HEG- and (**d**) TEG-based spacer-CE phosphoramidites, respectively used for **bisV7t1HEG2** and **bisV7t1TEG2D**.

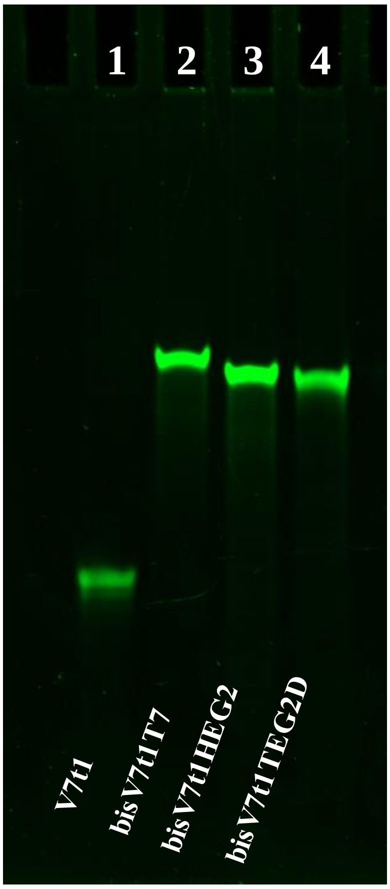

Figure S3. 20 % polyacrylamide denaturing gel electrophoresis (8 M urea) at 9 μM sample concentration, run at constant 200 V at r.t. for 3.5 h in TBE 1X as running buffer. Lane 1: V7t1; lane 2: **bisV7t1T7**; lane 3: **bisV7t1HEG2**; lane 4: **bisV7t1TEG2D**.

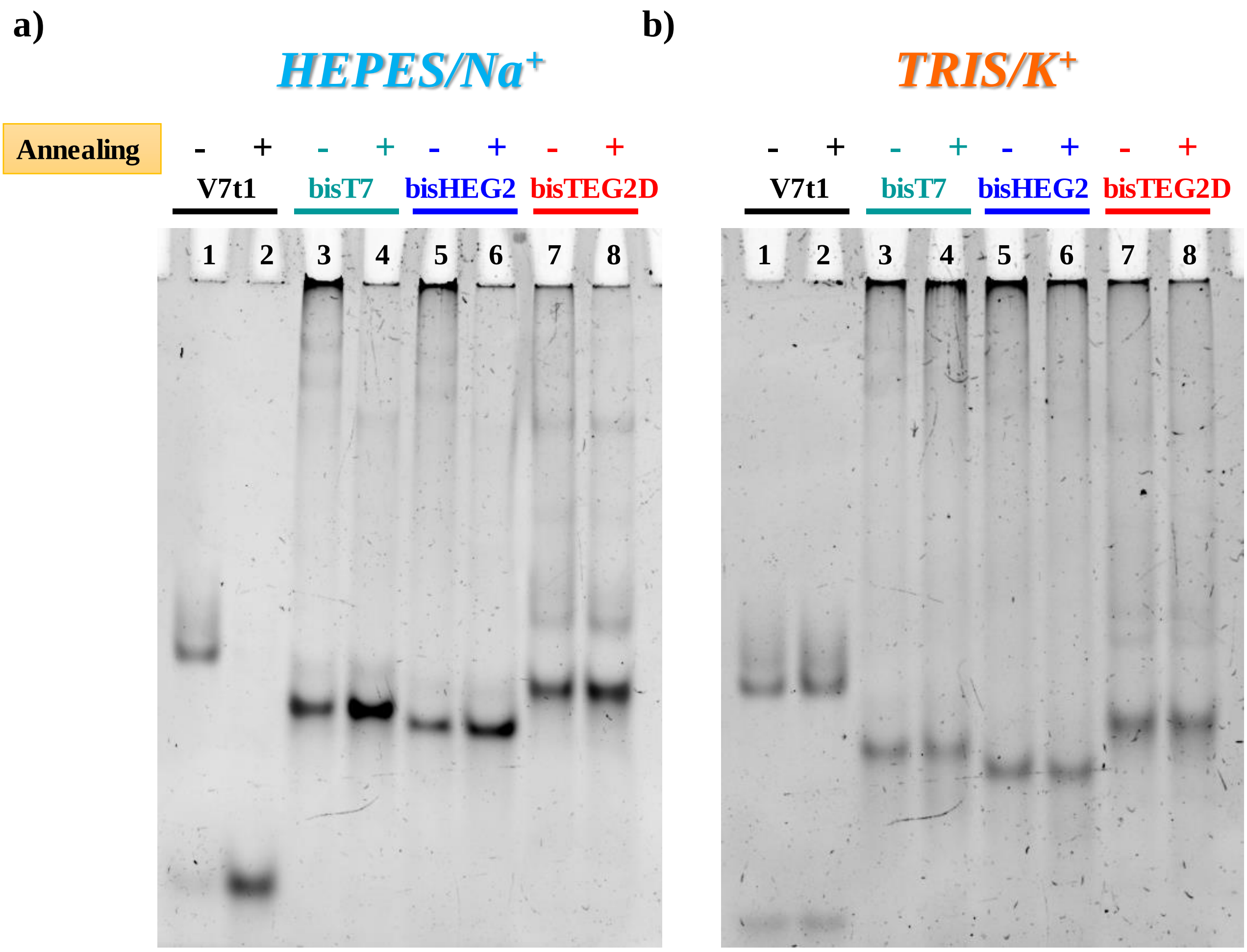

Figure S4. 10 % polyacrylamide gel electrophoresis under native conditions of V7t1 and its covalent dimers (here indicated for simplicity as bisT7, bisHEG2, bisTEG2D) in both N.A. (-) and A. (+) form at 4 μM concentration in the selected HEPES/ Na^+ (**a**) and TRIS/K^+ (**b**) buffer solutions. Gels were run at constant 70 V at r.t. for 1.75 h (**a**) and 2 h (**b**) in TBE 1X as running buffer.

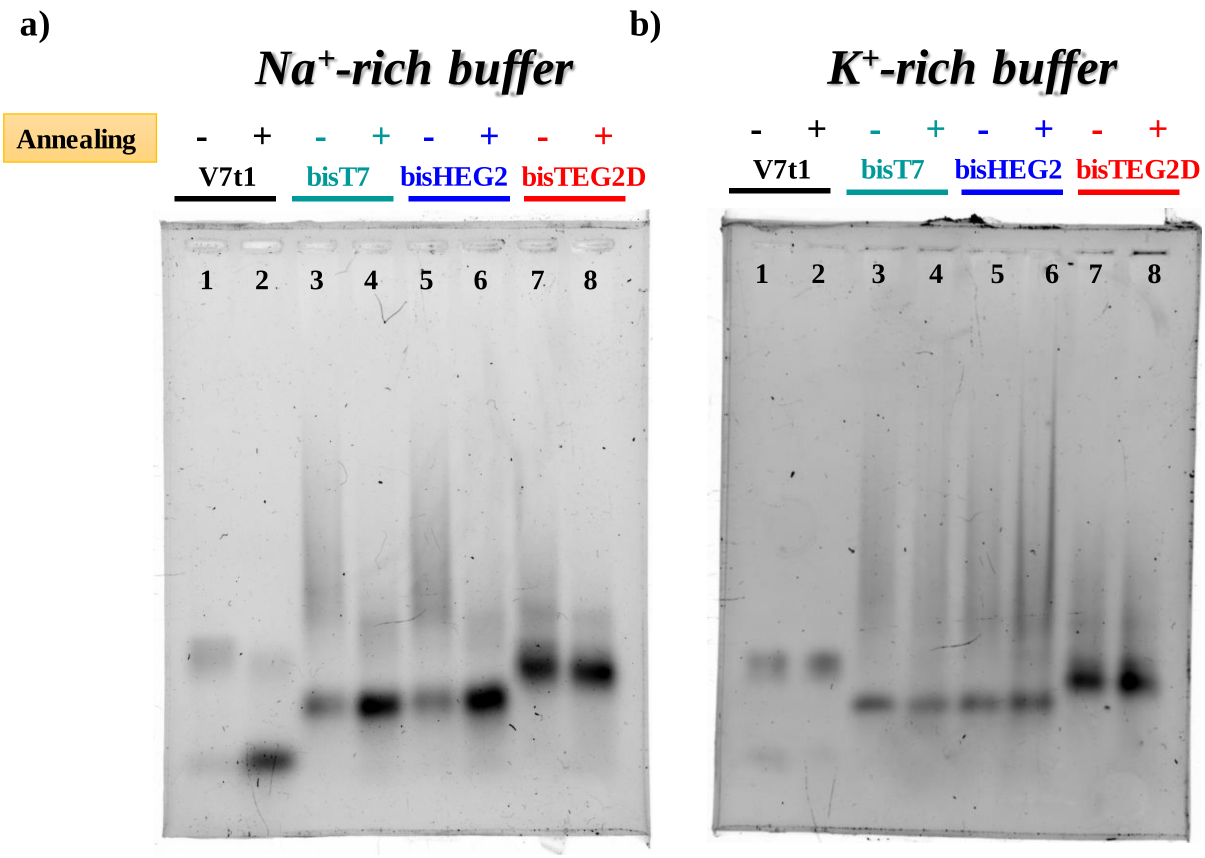

Figure S5. 2 % agarose gel electrophoresis under native conditions of V7t1 and its covalent dimeric analogues (here indicated as **bisT7**, **bisHEG2**, **bisTEG2D)** in both N.A. (−) and A. (+) form at 4 μM concentration in the amine-free 150 mM NaCl (pH = 7.4), as Na^+-rich buffer (**a**) and 100 mM KCl (pH = 7.3), as K^+-rich buffer (**b**) buffer solutions. Gels were run at constant 60 V at r.t. for 2 h in TBE 1X as running buffer.

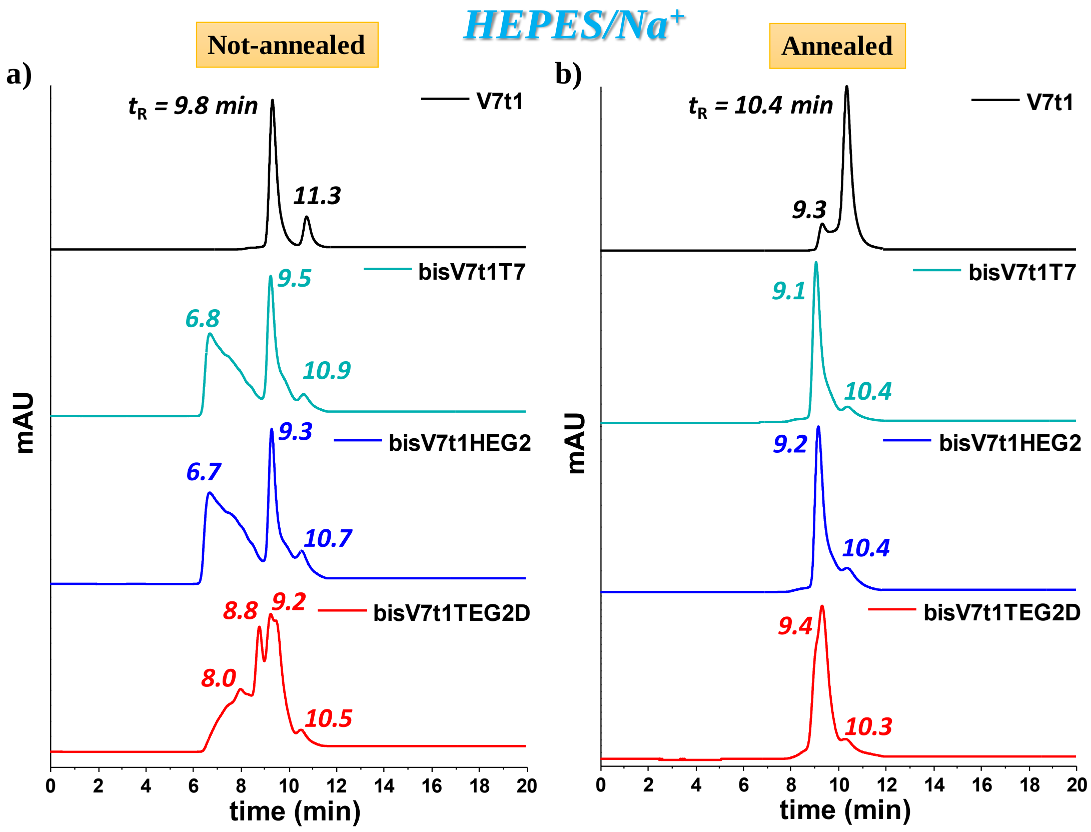

Figure S6. Size exclusion HPLC analysis of V7t1 (black line) and **bisV7t1T7**, **bisV7t1HEG2** and **bisV7t1TEG2D** (green, blue and red lines, respectively) in both N.A. (**a**) and A. (**b**) form in the selected HEPES/Na^+ buffer at 2 μM concentration. On each peak, the observed retention time (t_R) is also reported. The error associated with the t_R determination is within ± 5 %.

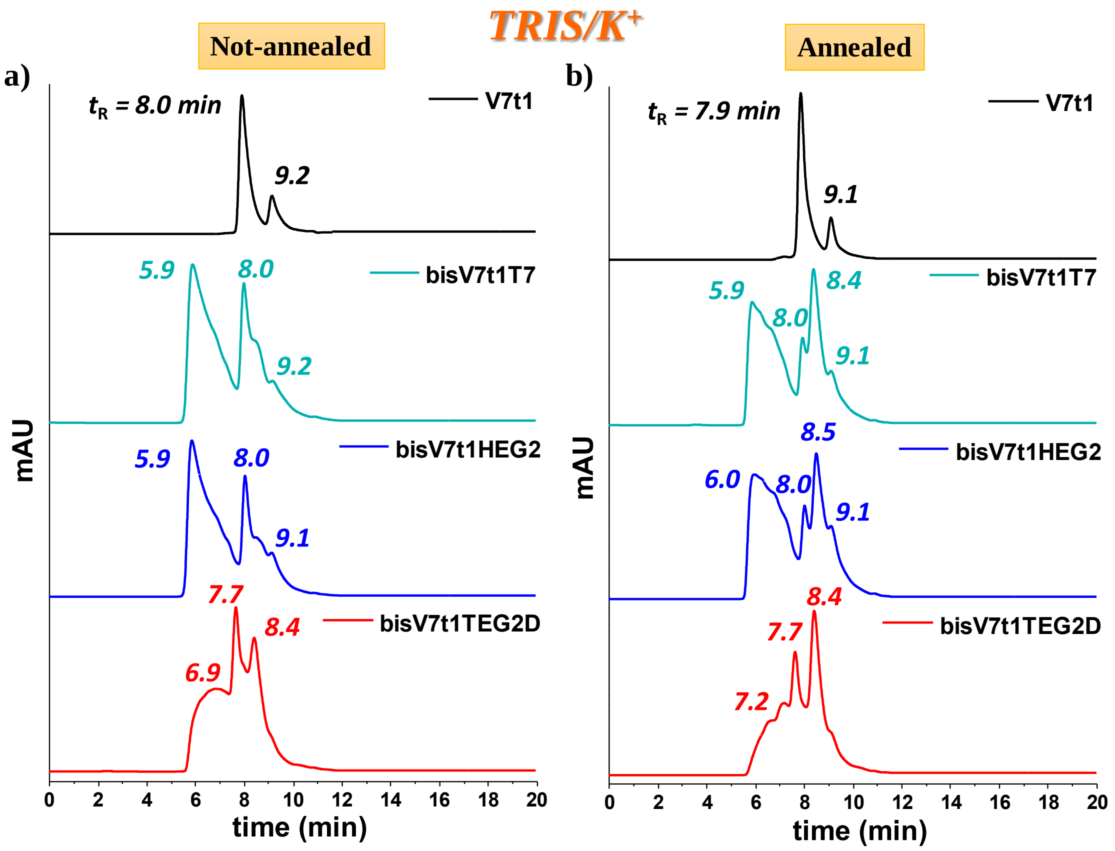

Figure S7. Size exclusion HPLC analysis of V7t1 (black line) and **bisV7t1T7**, **bisV7t1HEG2** and **bisV7t1TEG2D** (green, blue and red lines, respectively) in both N.A. (**a**) and A. (**b**) form in the selected TRIS/K^+ buffer at 2 μM concentration. On each peak, the observed retention time (t_R) is also reported. The error associated with the t_R determination is within ± 5%.

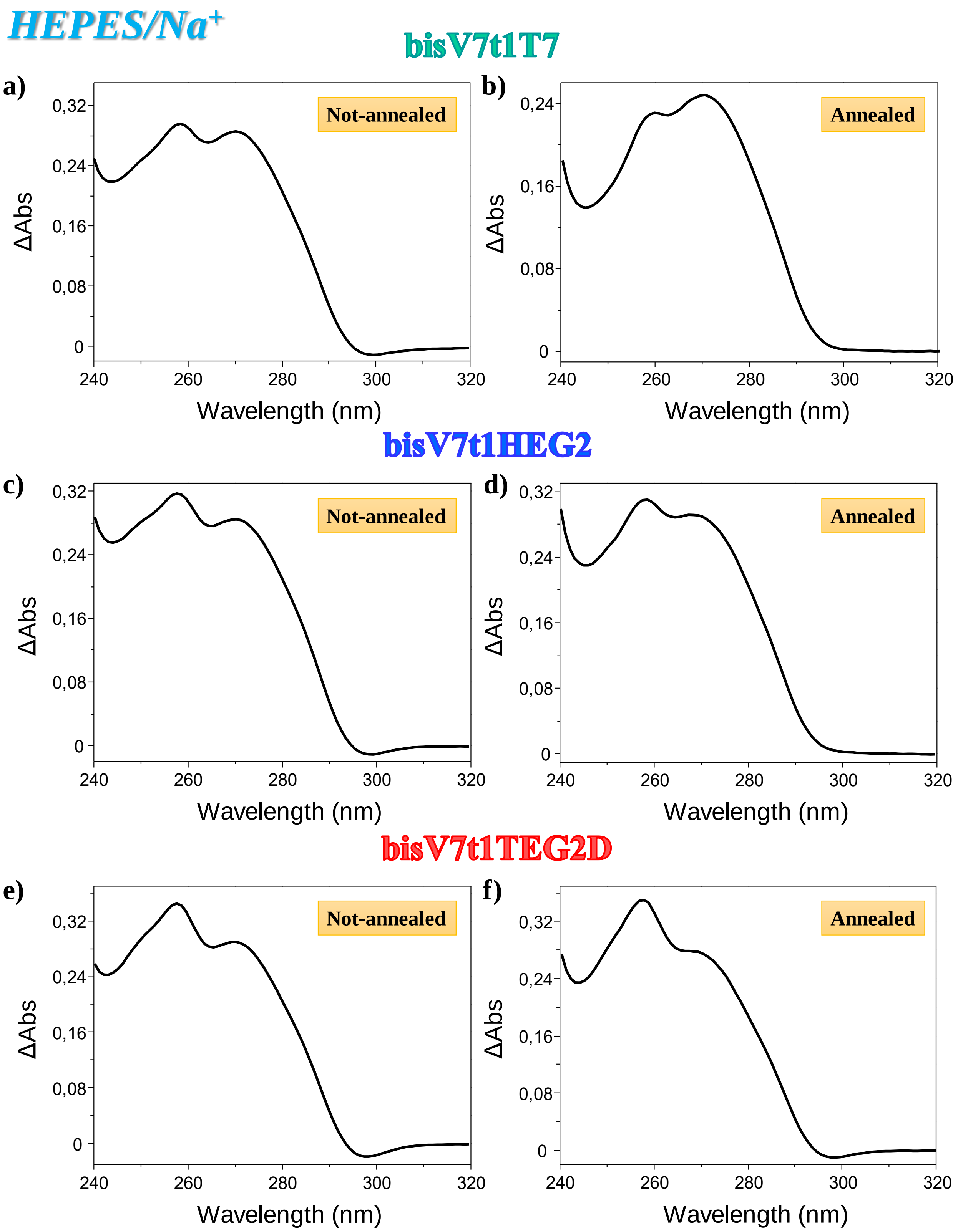

Figure S8. Thermal difference spectra (TDS) profiles of covalent V7t1 dimers, in both N.A. and A. form at 2 μM concentration in the selected HEPES/Na^+ buffer solution, resulting from the subtraction of the 15 °C spectrum from the 90 °C one.

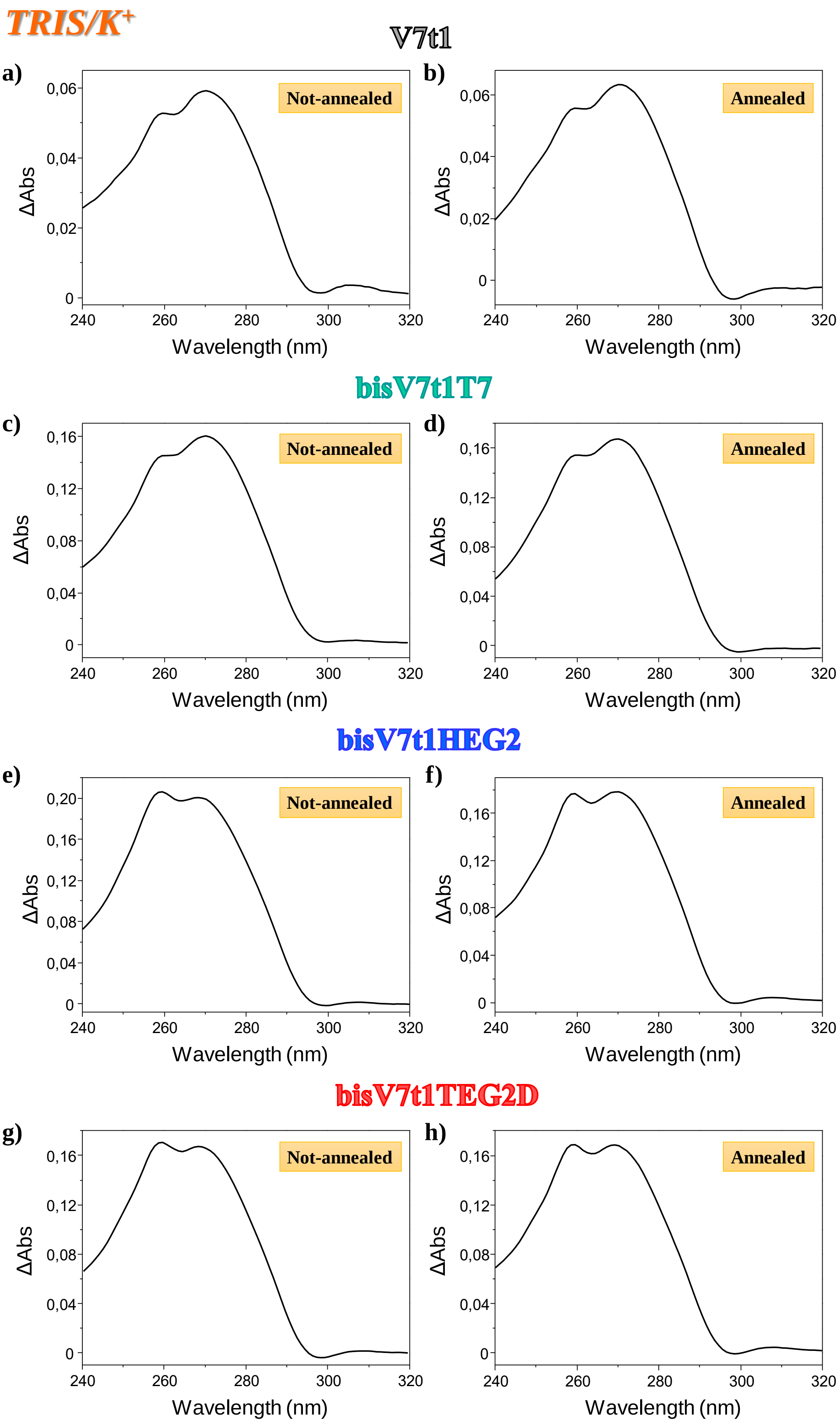

Figure S9. Thermal difference spectra (TDS) profiles of V7t1 and covalent V7t1 dimers, in both N.A. and A. form at 2 μM concentration in the selected TRIS/K^+ buffer solution, resulting from the subtraction of the 15 °C spectrum from the 90 °C one.

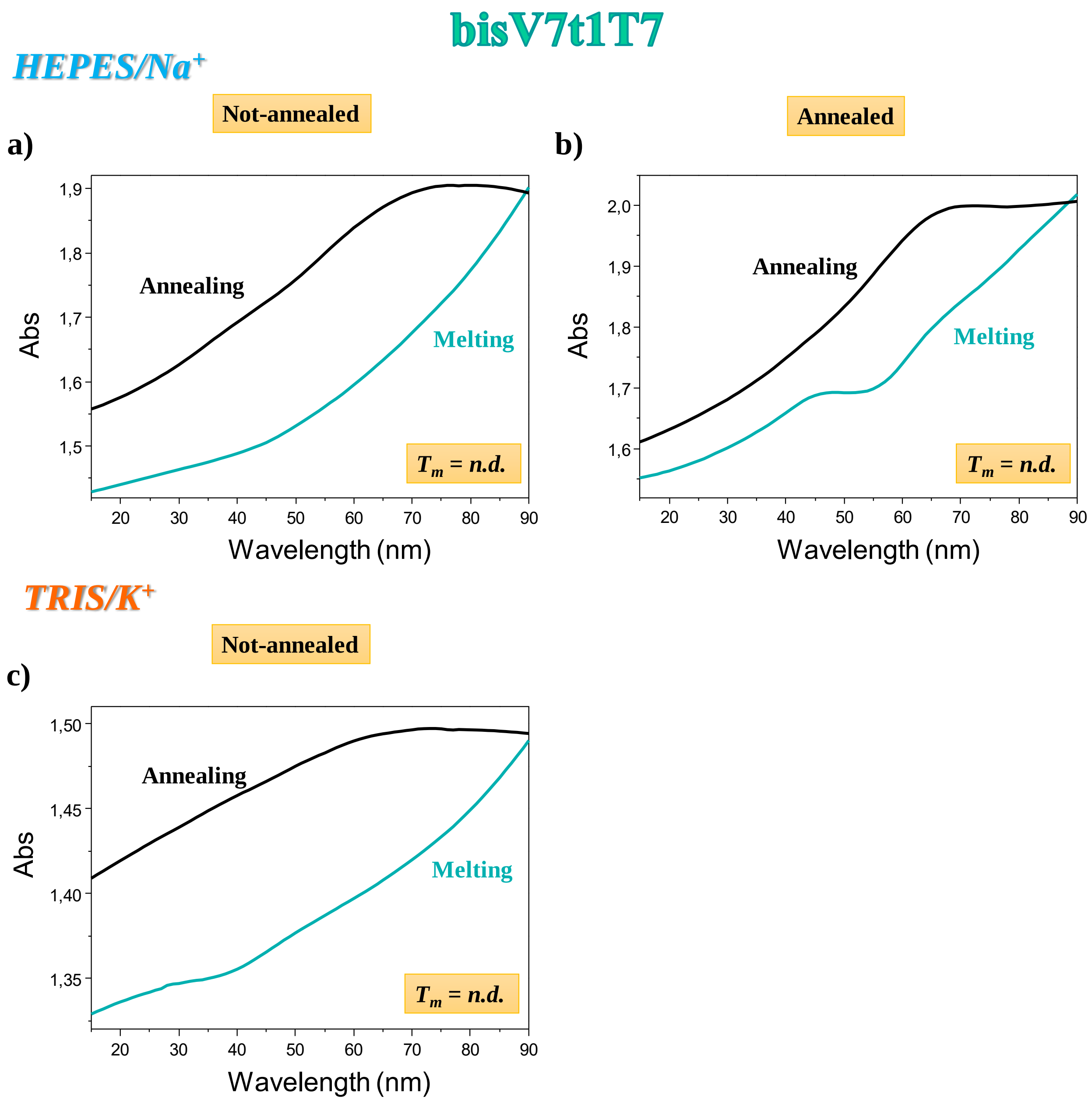

Figure S10. UV analysis on **bisV7t1T7** at 2 μM concentration in the selected HEPES/Na^+ (**a**, **b**) or TRIS/K^+ buffer solution in both N.A. (**a**, **c**) and A. (**b**) form: overlapped UV-melting and UV-annealing profiles (green and black lines, respectively) recorded at 260 nm using a scan rate of 1 °C/min. n.d. = not determined.

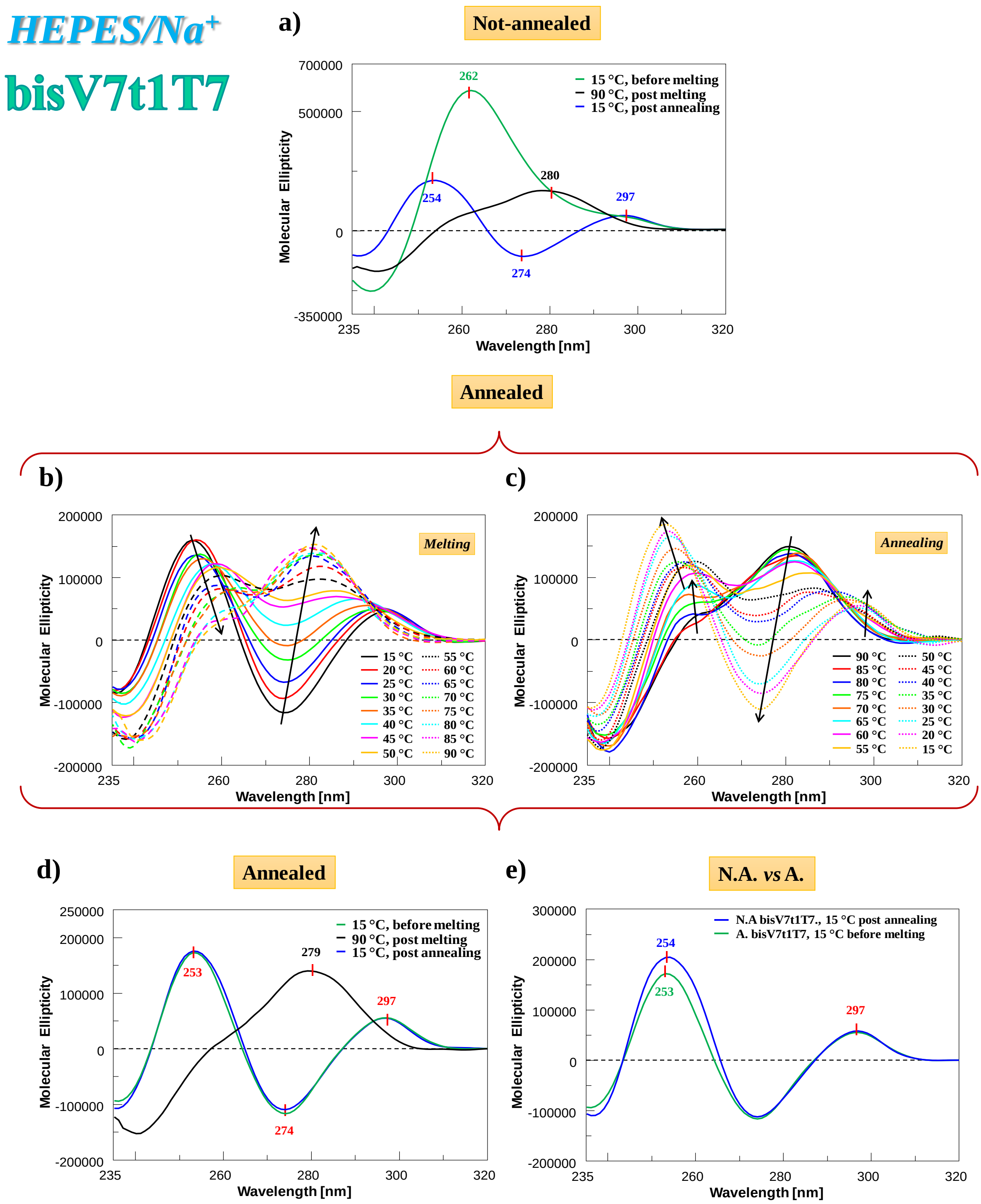

Figure S11. CD analysis performed on **bisV7t1T7** at 2 μM concentration in the selected HEPES/Na+ buffer solution in both N.A. and. A. form. Overlapped CD spectra of: (**a**) N.A. **bisV7t1T7** at 15 °C before melting, 90 °C after melting and 15 °C after annealing (green, black and blue lines, respectively); A. **bisV7t1T7** every 5 °C during the melting (**b**) and annealing (**c**) processes; (**d**) A. **bisV7t1T7** at 15 °C before melting, 90 °C after melting and 15 °C after annealing (green, black and blue lines, respectively); **e**) N.A. **bisV7t1T7** at 15 °C after annealing and A. **bisV7t1T7** at 15 °C before melting (blue and green lines, respectively). Arrows in panels **c** and **d** indicate the evolution of the CD signal over time.

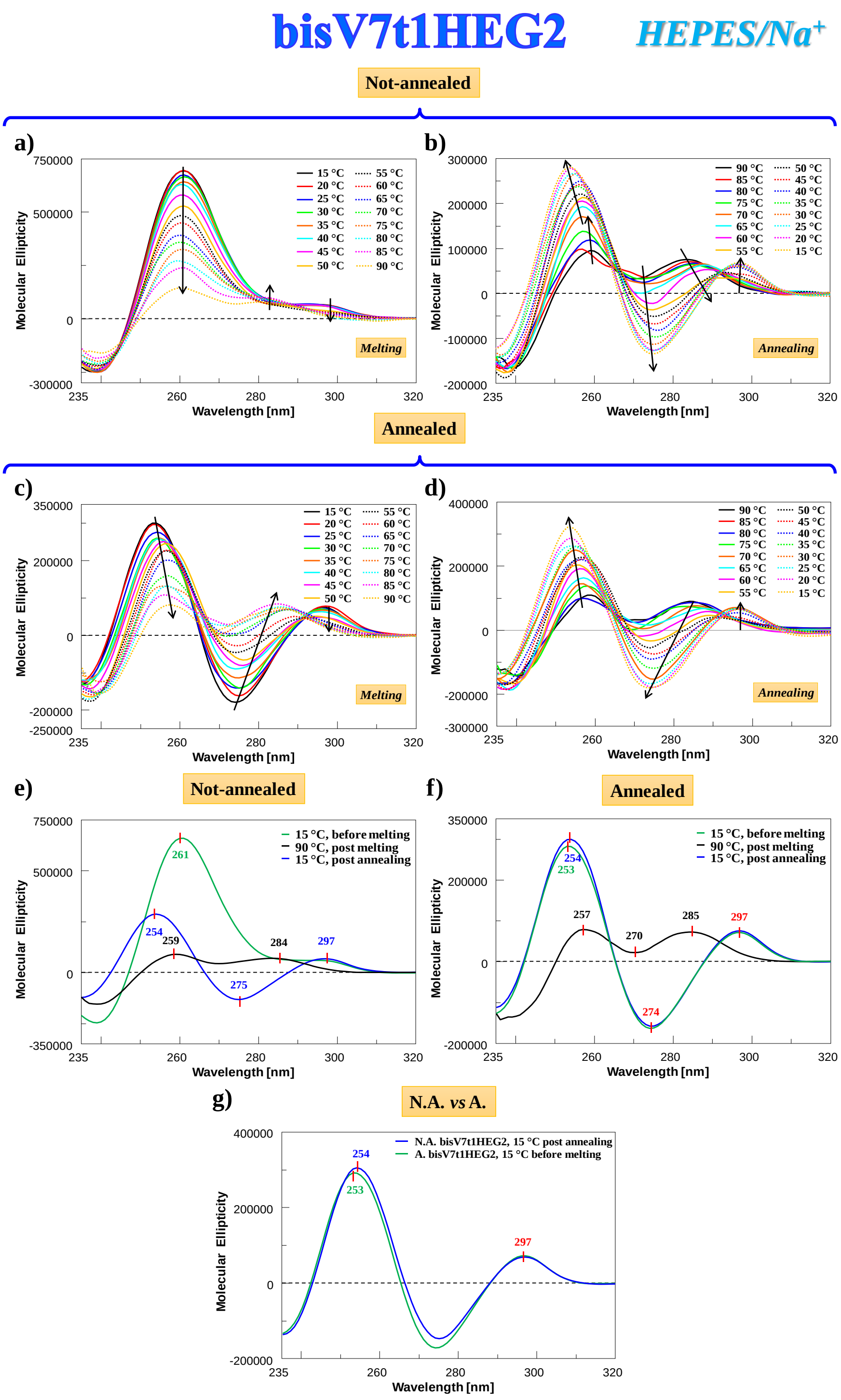

Figure S12. CD analysis performed on **bisV7t1HEG2** at 2 μM concentration in the selected HEPES/Na+ buffer solution in both N.A. and. A. form. Overlapped CD spectra of: N.A. **bisV7t1HEG2** recorded every 5 °C during the melting (**a**) and annealing (**b**) processes; A. **bisV7t1HEG2** recorded every 5 °C during the melting (**c**) and annealing (**d**) processes; **e**) N.A. **bisV7t1HEG2** at 15 °C before melting, 90 °C after melting and 15 °C after annealing (green, black and blue lines, respectively); **f**) A. **bisV7t1HEG2** at 15 °C before melting, 90 °C after melting and 15 °C after annealing (green, black and blue lines, respectively); **g)** N.A. **bisV7t1HEG2** at 15 °C after annealing and A. **bisV7t1HEG2** at 15 °C before melting (blue and green lines, respectively). Arrows in panels **a-d** indicate the evolution of the CD signal over time.

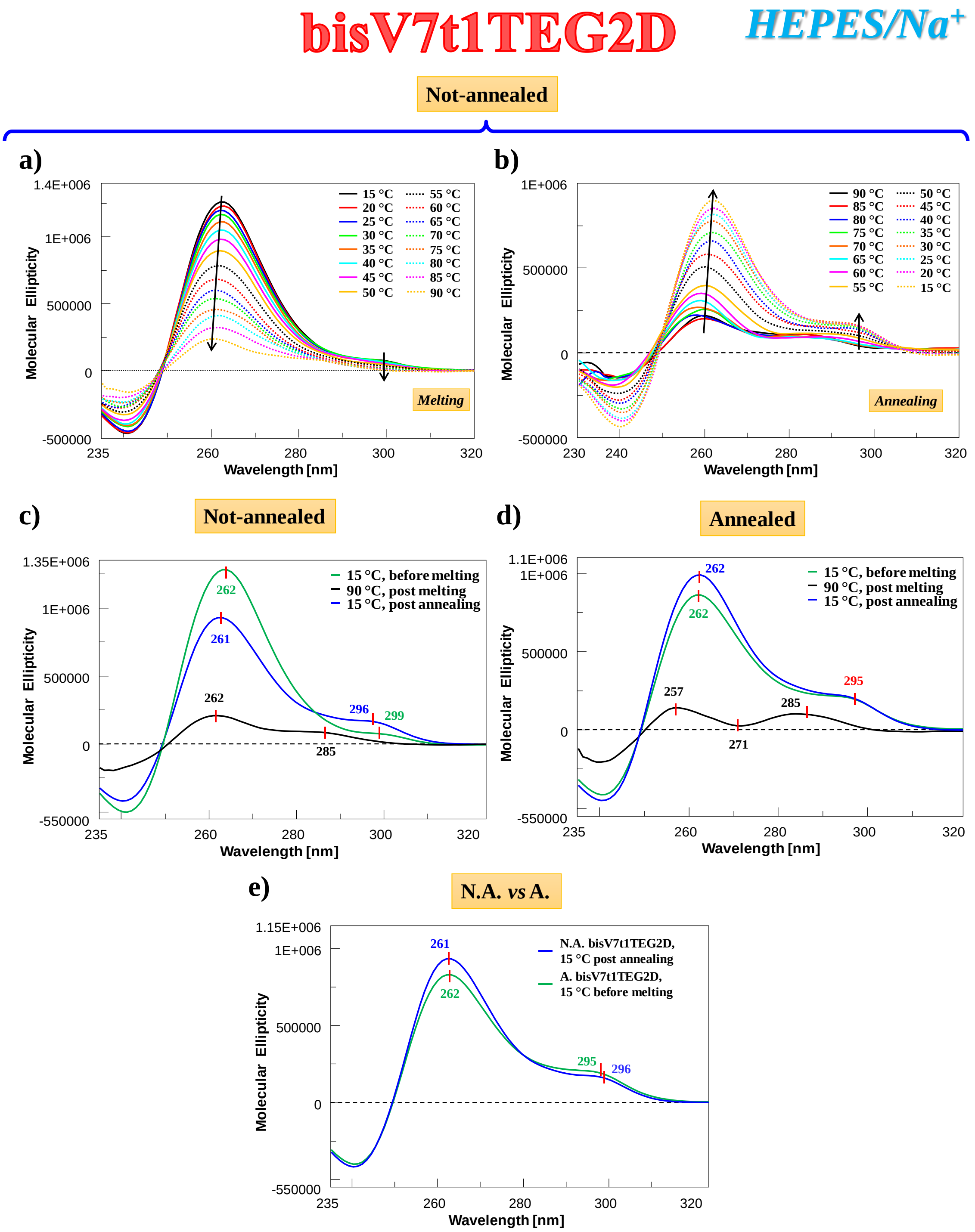

Figure S13. CD analysis performed on **bisV7t1TEG2D** at 2 μM concentration in the selected HEPES/Na+ buffer solution in both N.A. and. A. form. Overlapped CD spectra of: N.A. **bisV7t1TEG2D** recorded every 5 °C during the melting (**a**) and annealing (**b**) processes; **c**) N.A. **bisV7t1TEG2D** at 15 °C before melting, 90 °C after melting and 15 °C after annealing (green, black and blue lines, respectively); **d**) A. **bisV7t1TEG2D** at 15 °C before melting, 90 °C after melting and 15 °C after annealing (green, black and blue lines, respectively); **e**) N.A. **bisV7t1TEG2D** at 15 °C after annealing and A. **bisV7t1TEG2D** at 15 °C before melting (blue and green lines, respectively). Arrows in panels **a** and **b** indicate the evolution of the CD signal over time.

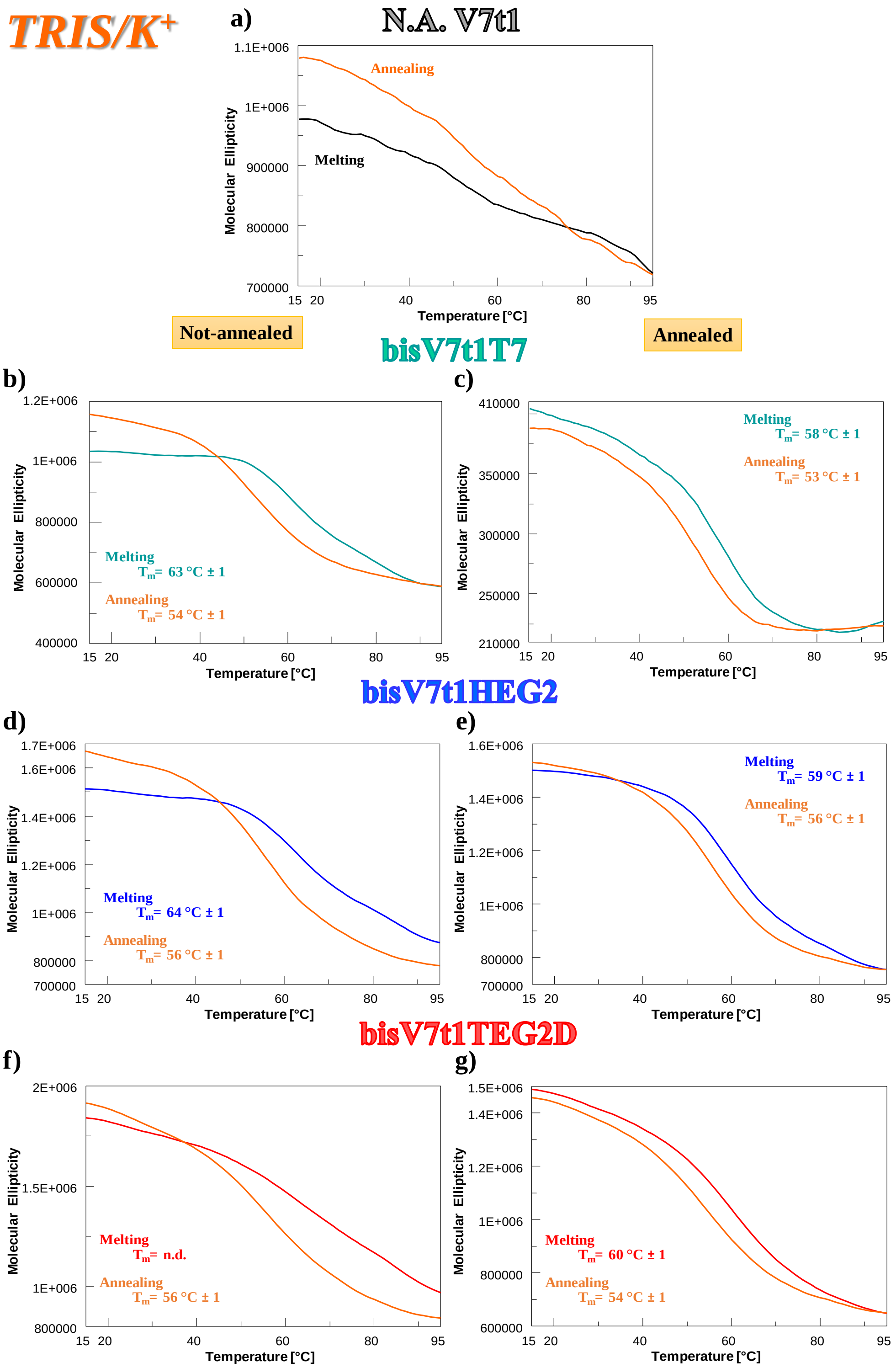

Figure S14. CD analysis performed on V7t1 and its covalent V7t1 dimers at 2 μM concentration in the selected TRIS/K^+ buffer solution in both N.A. and. A. form. CD-melting and -annealing profiles of: (**a**) N.A. V7t1, recorded at 263 nm; (**b**) N.A. and (**c**) A. **bisV7t1T7**, recorded at 264 and 268 nm, respectively; (**d**) N.A. and (**e**) A. **bisV7t1HEG2**, both recorded at 263 nm; (**f**) N.A. and (**g**) A. **bisV7t1TEG2D**, recorded at 263 and 264 nm, respectively. All the annealing profiles are depicted as orange lines while melting curves are represented as black, green, blue and red lines respectively for **V7t1**, **bisV7t1T7**, **bisV7t1HEG2** and **bisV7t1TEG2D**. All the thermal profiles were recorded using a scan rate of 1 °C/min. n.d. = not determined.

Table S1. Melting temperature values obtained by CD-monitored thermal denaturation experiments for heating and cooling profiles of V7t1 and the here investigated covalent V7t1 dimers in the selected HEPES/Na^+ and TRIS/K^+ buffer solutions (n.d. = not determined).

	HEPES/Na^+		TRIS/K^+	
	CD T_m (°C) ± 1			
	Not-annealed	Annealed	Not-annealed	Annealed
	Melting/Annealing	Melting/Annealing	Melting/Annealing	Melting/Annealing
V7t1	n.d. / n.d.	50 / 48	n.d. / n.d.	n.d. / n.d.
bisV7t1T7	n.d. / n.d.	n.d. / n.d.	63 / 54	58 / 53
bisV7t1HEG2	n.d. / n.d.	n.d. / n.d.	64 / 56	59 / 56
bisV7t1TEG2D	n.d. / 52	55 / 54	n.d. / 56	60 / 54

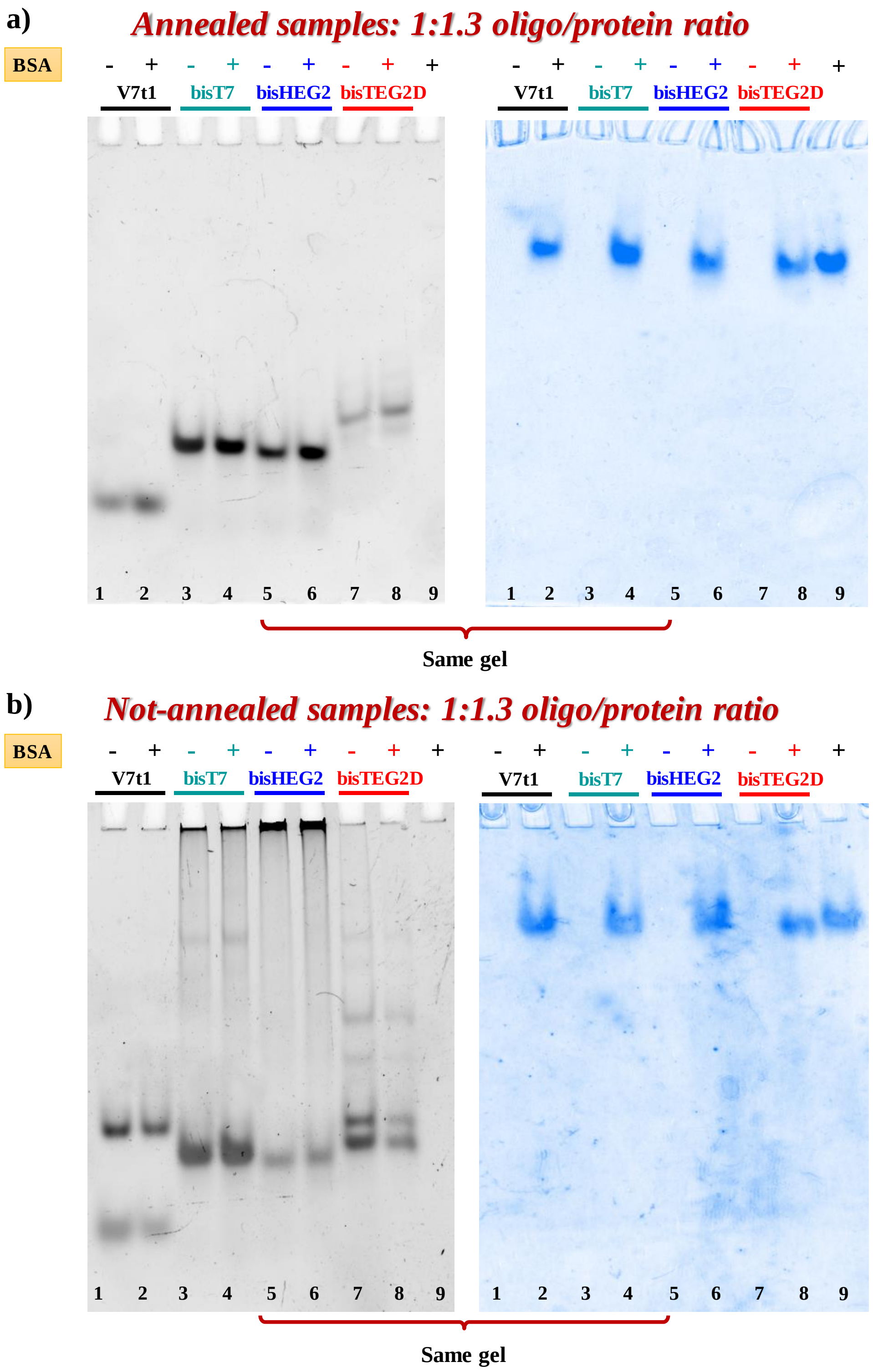

Figure S15. Native 7 % EMSA of A. (**a**) and N.A. (**b**) V7t1 and covalent V7t1 dimers incubated in the presence (+) or absence (–) of BSA. GelGreen- and Coomassie-stained gels (left and right, respectively). 30 pmol of each aptamer were incubated with 40 pmol of the protein in a final volume of 9 μL in the selected HEPES/Na^+ buffer, thus obtaining a final 1:1.3 oligo/protein ratio. Gels were run at constant 45 V for 2.3 h at r.t. in TAE 1X buffer.
